# Nanoparticles and their significant risks: a comprehensive review of toxicological and regulatory perspectives

**DOI:** 10.1186/s12951-026-04801-8

**Published:** 2026-07-30

**Authors:** Beata Skonieczna, Natalia Nazarko, Ewelina Godlewska, Natalia Wasiluk, Ewa Katarzyna Czech, Agnieszka Zofia Wilczewska, Elżbieta Regulska, Halina Car, Katarzyna Niemirowicz-Laskowska

**Affiliations:** 1 Department of Experimental Pharmacology, Medical University of Bialystok, Szpitalna 37, Bialystok, 15-295 Poland; 2 Faculty of Law, University of Bialystok, Adama Mickiewicza 1, Bialystok, 15-213 Poland; 3 Faculty of Chemistry, University of Bialystok, Ciolkowskiego 1K, Bialystok, 15-245 Poland; 4 Faculty of Pharmacy, University of Castilla-La Mancha, Calle Almansa 14 – Edif. Bioincubadora, Albacete, 02008 Spain

**Keywords:** Nanoparticles, Drug carriers, Toxicity, Cellular effects, Immunological effects, Liver enzymatic activity, Gut microbiome, Reproductive toxicity, Animal studies, Human studies, Regulatory aspects

## Abstract

**Background:**

Nanoparticles are increasingly important as drug carriers due to their ability to enhance solubility, stability, and selective delivery of therapeutic agents, enabling accumulation at disease sites, interaction with cell-surface receptors, and penetration of biological barriers such as the blood-brain barrier. Despite these numerous advantages, their potential toxicity remains a key obstacle to safe clinical use. Emerging evidence suggests that nanoparticles may alter the composition of the gut microbiome, potentially affecting metabolism, immune homeostasis, neurobehavioral pathways, and reproductive function by influencing hormonal regulation and fertility.

**Aim of the review:**

This review provides a detailed analysis of the toxicological aspects of nanoparticles, including cytotoxicity, oxidative stress, potential genotoxicity, and effects on immune responses such as inflammation and immunosuppression. In vivo biodistribution, organ accumulation, and effects on liver enzymes are also discussed, emphasizing the role of the liver in drug metabolism and the potential risk of impaired detoxification. Additionally, the review examines the effects of nanoparticles on the gut microbiome and reproductive system. It emphasizes their capacity to disrupt the microbial balance, the intestinal barrier integrity, and reproductive hormone homeostasis. We discuss regulatory issues for nanotechnology-based medicinal products, emphasizing the importance of standardized testing protocols and long-term safety assessments. Finally, future perspectives and developmental directions for nanoparticles have been highlighted.

**Conclusion:**

This review highlights the need for a comprehensive understanding of nanoparticle toxicity mechanisms, which is crucial for their safe design and further development in nano-based therapy.

**Graphical Abstract:**

**Figure:**
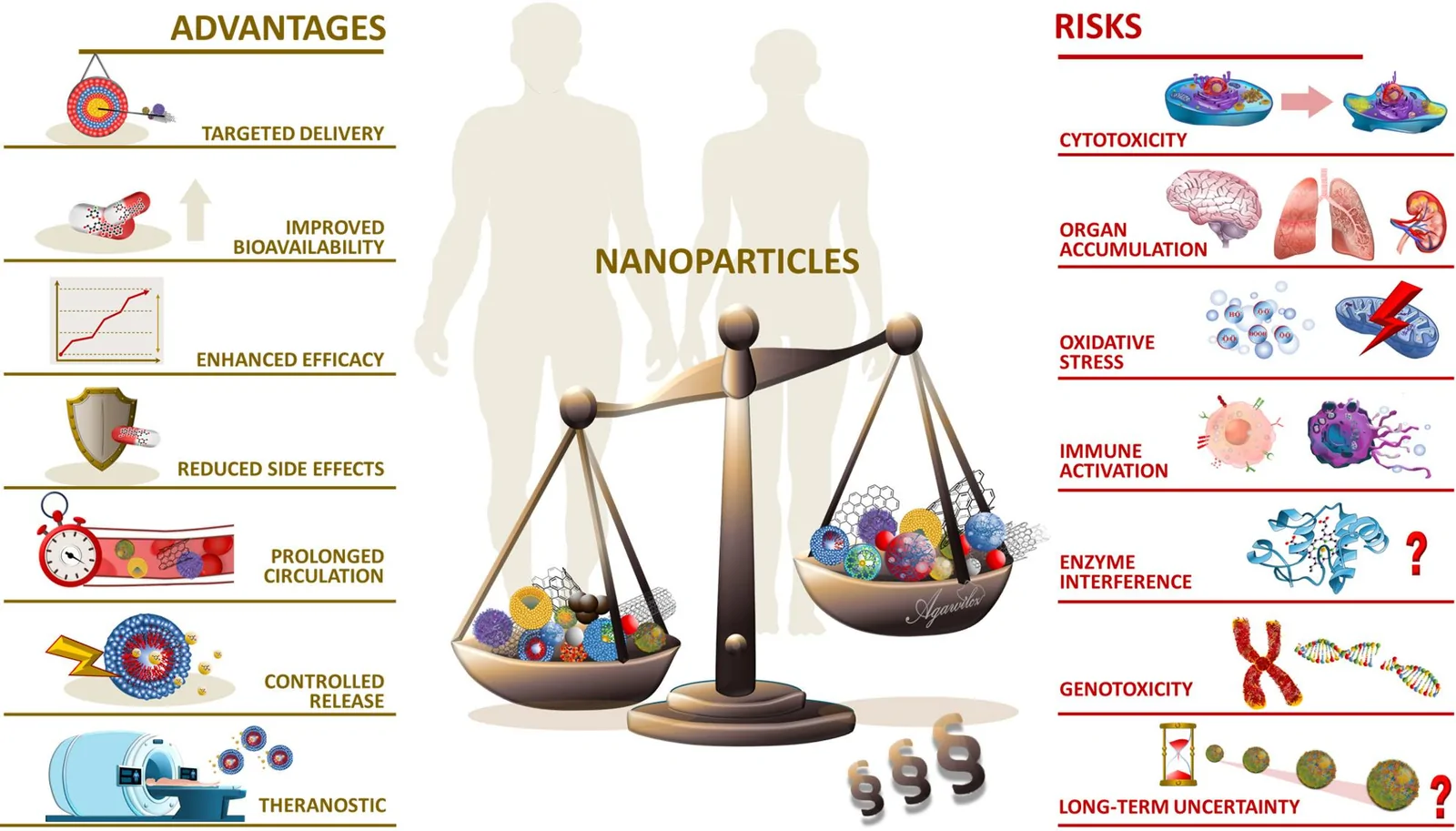

## Introduction

Could nanoparticles, often considered the future of medicine, also represent an important challenge for their safe and effective clinical application? Nanomaterials play a key role in the development of modern drug delivery systems. They enable the development of therapies that precisely target specific parts of the body. Nanomaterials can cross biological barriers, e.g., the blood-brain barrier, and prolong the circulation time of pharmaceuticals in the body [1, 2]. Nanoparticles are formed from both organic and inorganic materials. They can be composed from dendrimers, polymers, lipids, carbon nanotubes, quantum dots, and metal oxides and may differ substantially in their size, shape, and surface properties, which can significantly influence their biological interactions and toxicity profiles. When combined with therapeutic substances, they can form liposomes, micelles, microspheres, polyplexes, conjugates, or polymer nanoparticles. These structures ensure more effective drug delivery to the site of disease and reduce toxicity to healthy tissues [1–3].

However, alongside this potential, there are serious safety concerns. The most thoroughly studied are inorganic nanocarriers, including silver nanoparticles, zinc oxide, titanium dioxide, and carbon nanomaterials, whose toxicity depends on size, shape, composition, and aggregation [4]. The effects of their accumulation in the body, their interaction with the immune system, their ability to generate reactive oxygen species (ROS), as well as their biodistribution and metabolism, remain incompletely understood [1]. Small nanoparticles may also interact with enzymes such as Cytochrome P450 in the liver, thereby disrupting detoxification mechanisms and leading to the accumulation of nanocarriers in the body [5]. Surface charge also remains an important factor, as it affects their uptake by hepatocytes or macrophages, and increases the risk of interaction with metabolic enzymes, as shown by Elci et al. [6]. This issue is further exacerbated by the fact that most of the nanocarrier toxicity studies are conducted at the preclinical stage, thereby limiting access to detailed data and hindering comprehensive risk assessment for clinical applications [7]. Tissues particularly exposed to their effects include the sites of drug-carrier complex administration, blood morphogenic elements, vessel walls, and detoxification organs such as the liver, kidneys, spleen, and immune system.

In recent years, it has been evidenced that nanoparticle toxicity affects the gut microbiome, a flora of a key role in maintaining homeostasis. Nanomaterials can interfere with the composition and function of the microbiome. This can lead to declines in microbial diversity (α- and β-diversity) [8–10], reductions in probiotic bacteria (e.g., *Lactobacillus*, *Bifidobacterium*, and *Faecalibacterium*) [11–13], and bacteria that produce short-chain fatty acids (SCFAs) [14–16]. Moreover, NPs can increase the number of potentially pro-inflammatory species, e.g., *Proteobacteria*, *Enterobacteriaceae*, and *Desulfovibrio.* This, in turn, may increase intestinal permeability and impair SCFA metabolism, thereby promoting dysbiosis and chronic inflammation [10, 14, 15]. Furthermore, some nanomaterials can penetrate the tissues of the reproductive system. Preclinical studies have shown their accumulation in the testes, ovaries, and placenta. This accumulation may result in impaired spermatogenesis, oocyte damage, changes in sex hormone levels, and increased oxidative stress [16–21]. Some nanoparticles can cross the blood-testis and placental barriers, posing a potential risk to fertility and embryonic development [22–27].Therefore, a key challenge in nanomedicine nowadays should focus on a complete understanding of the mode of action and evaluation of the toxicity of nanocarriers. The safety of these substances can only be determined by systematically assessing their impact on the body (cytotoxicity, oxidative stress, interactions with detoxification enzymes). It will then be possible to safely exploit their enormous therapeutic potential while accounting for the risk of accumulation in organs. The aim of this paper is to present the current state of knowledge on the side effects of nanoparticles mainly used as drug carriers and to indicate the directions of research necessary to ensure their safe use in medicine.

In this review, we discuss the toxicity of nanoparticles, focusing on their effects on cellular systems, immune function, and hepatic enzyme activity. Particular attention is given to their interactions with the intestinal microbiome and potential reproductive toxicity, which represent important yet still underexplored areas in nanotoxicology. We critically evaluated both preclinical animal studies and the available clinical evidence to provide an integrated perspective on nanoparticle safety. In addition, relevant regulatory aspects were considered in order to contextualize current safety assessment frameworks and support a comprehensive evaluation of nanomaterial-associated risks. Finally, future perspectives, including key challenges and potential research directions, have been proposed to highlight current knowledge gaps and guide further development in the field.

In contrast to previous reviews that have focused predominantly on individual toxicological mechanisms or specific nanoparticle categories, the present review offers a comprehensive overview of the biological, toxicological, and regulatory challenges associated with pharmaceutical nanocarriers. Particular emphasis is placed on emerging and currently underrepresented aspects of nanosafety, including nanoparticle-induced alterations of the gut microbiota, reproductive toxicity, and potential effects on germ-cell integrity. Furthermore, this review integrates mechanistic toxicology with translational and regulatory perspectives in order to provide a broader understanding of the challenges associated with the safe clinical implementation of nanomedicine.

It is important to acknowledge that in numerous studies assessing nanoparticle toxicity, the observed biological effects are attributed to the entire nanosystem rather than to its individual components. In most of experimental designs, nanoparticles are evaluated as integrated constructs without systematically distinguishing between the contribution of the core material, surface functionalization, loaded therapeutic agents, degradation products, or potential impurities. This limitation makes it difficult to unequivocally attribute specific toxic effects to a single component of the nanocarrier. Furthermore, variability in physicochemical characterization, together with the lack of standardized testing protocols, further complicates cross-study comparisons.

Consequently, the interpretation of nanoparticle-induced toxicity should be approached with caution, and future studies should focus on more controlled experimental designs that would allow the individual contributions of nanosystem components to be more clearly distinguished.

The existing literature on this subject was compiled using PubMed and ScienceDirect. The search covered publications published between 2000 and 2025 and was performed using combinations of the following keywords: ‘drug nanocarriers’, ‘nanocarrier toxicity’, ‘nanocarrier side effects’, ‘nanoparticle toxicity’, ‘nanoparticle side effects’, ‘nanomedicine toxicity’, ‘immunotoxicity of nanoparticles’, ‘reproductive toxicity of nanoparticles’, and ‘gut microbiome toxicity of nanoparticles’, ‘biomedical applications of nanoparticles’, ‘nanomedicine’, ‘drug delivery systems’, ‘theranostics’, ‘lipid nanoparticles’, ‘polymeric nanoparticles’, ‘metal nanoparticles’, ‘magnetic nanoparticles’, ‘core-shell nanoparticles’, ‘cancer nanotherapy’, ‘targeted drug delivery’, ‘nanoparticles in diagnostics and imaging’, ‘biosensors’, ‘antibacterial nanoparticles’, ‘antifungal nanoparticles’, ‘FDA-approved nanotherapeutics’, ‘nanomedicine regulation’, ‘nanoparticle characterization’, ‘protein corona’, ‘machine learning in nanomedicine’, ‘AI in nanotoxicology’, ‘green synthesis of nanoparticles’, ‘continuous manufacturing of nanomaterials’, ‘personalized nanomedicine’, ‘stimuli-responsive nanocarriers’, and ‘smart nanocarriers’. We prioritized original research articles that had undergone peer review, as well as reviews and regulatory documents. These articles, reviews, and documents addressed the biological effects, biomedical applications, translational challenges, regulatory aspects, manufacturing strategies, and toxicological properties of nanoparticles used in drug delivery systems. Special attention was also given to studies concerning clinically approved nanotherapeutics, targeted drug delivery, theranostic applications, nanoparticle characterization, artificial intelligence-assisted nanomedicine design, and future perspectives for clinical translation. Publications not directly related to nanocarrier-associated toxicity or lacking sufficient toxicological relevance were excluded from the analysis.

## Application of nanoparticles in biomedicine

The field of nanotechnology has effectively blurred the boundaries between the biological and physical sciences by introducing innovative nanostructures and nanophase systems into numerous research disciplines. Of particular interest is its application in nanomedicine and drug delivery system design, which currently represent one of the key areas of development in this field [28]. Recent scientific analyses have confirmed the significant role of nanotechnology in addressing global challenges across medicine and environmental protection. The utilization of nanomaterials offers new possibilities in environmental remediation and the conservation of natural resources [29].

### History of nanotechnology

Richard Feynman is considered the father of modern nanotechnology. In 1959, he presented the concept of controlling individual atoms and molecules, although he did not yet use the term “nanotechnology” [30]. The term was introduced in 1974 by Norio Taniguchi [31]. The subsequent development of nanotechnology was closely associated with advances in materials engineering, chemistry, and molecular biology, which enabled the design and characterization of nanoscale materials [31, 32].

Among the first nanoscale structures used in medicine were liposomes, described by Alec D. Bangham and R. W. Horne in 1964 [33]. The development of drug delivery systems was based on the progressive implementation of innovative biomaterials, beginning with the introduction of the first polymers for the controlled release of macromolecules. Another breakthrough occurred in the 1980s with the development of pH-responsive systems and pioneering methods for targeting liposomes to specific cells. Towards the end of that decade, the concept of so-called “stealth liposomes” was introduced. Combined with PEGylation technology developed in the 1990s, this approach significantly prolonged the circulation time of nanocarriers in the body and paved the way for the commercialisation of the first nanomedicines [34].

Due to their biomimetic structure, which closely resembles that of biological membranes, liposomes have become the subject of intensive research as universal drug carriers, as described by Gregoriadis [35, 36]. Their unique structure enables the efficient encapsulation of compounds with diverse physicochemical properties, including both hydrophilic and lipophilic substances. Consequently, this improves the pharmacological profile of therapeutics and reduces their systemic toxicity.

### Contemporary nanomedicine

Over the past two decades, nanotechnology has become one of the most rapidly developing fields in biomedical science. This is primarily associated with the possibility of designing carriers capable of the controlled delivery of active substances to specific tissues and cells [32]. The ability of nanosystems to interact synergistically with cellular structures enables the effective integration of diagnostic and therapeutic functions [29]. The continuous development of nanomedicine offers several advantages over conventional pharmacotherapy, including improved therapeutic efficacy, enhanced bioavailability, a more favourable dose-response relationship, and increased treatment safety and personalization [32]. Such an integrated approach enables precise biological imaging, selective detection of cancer cells, and the implementation of targeted multimodal therapeutic strategies [29]. Currently, the development of multifunctional nanoparticle complexes capable of simultaneously delivering diagnostic and therapeutic agents directly to the disease site is considered one of the most promising research directions [32].

Nanomedicine is a rapidly evolving discipline that applies nanoscale technologies to medical biology, disease prevention, and modern therapeutic strategies. Its scope includes nanoscale structures, such as diagnostic nanosensors and intelligent nanorobots, as well as materials that can modulate intracellular processes. One of the most significant achievements in this field is the development of nanoparticle-based platforms integrating therapeutic functions with cancer imaging diagnostics [32]. The first generation of nanotherapy was based on lipid systems, including micelles and liposomes, which received approval from the U.S. Food and Drug Administration [28].

### Nanoparticles as drug delivery systems

Currently, lipid carriers are used to encapsulate inorganic nanostructures, such as gold and magnetite nanoparticles, thereby expanding their potential for targeted drug delivery and biomedical imaging. Furthermore, nanostructures protect active compounds from degradation in the gastrointestinal tract and enable the efficient delivery of poorly water-soluble drugs directly to target tissues. Owing to the use of natural endocytic absorption mechanisms, nanotechnology-based formulations demonstrate significantly higher oral bioavailability compared with conventional pharmaceutical formulations [28].

Metal nanoparticles also play an important role in modern nanotechnology, with applications in electronic systems, targeted drug delivery, genetic engineering, and environmental remediation. The ability to control the morphology, size, and surface properties of metallic nanoparticles enables their adaptation to specific biomedical and biological applications. The physicochemical properties of these structures, including surface plasmon resonance, vary depending on particle size and concentration, directly influencing their functionality in biological systems [29].

Modern nanoparticles are characterised by a complex, multilayered structural architecture comprising three interdependent layers. The innermost core forms the structural basis of the nanoparticle and is surrounded by a shell modified with ions, surfactants, polymers, or small molecules. The outer layer consists of ligands and peptides that functionalize the particle surface and confer specific biological properties. Such structural modifications considerably broaden the range of potential applications of nanostructures in biomedical and scientific research [29].

**Fig. 1:**
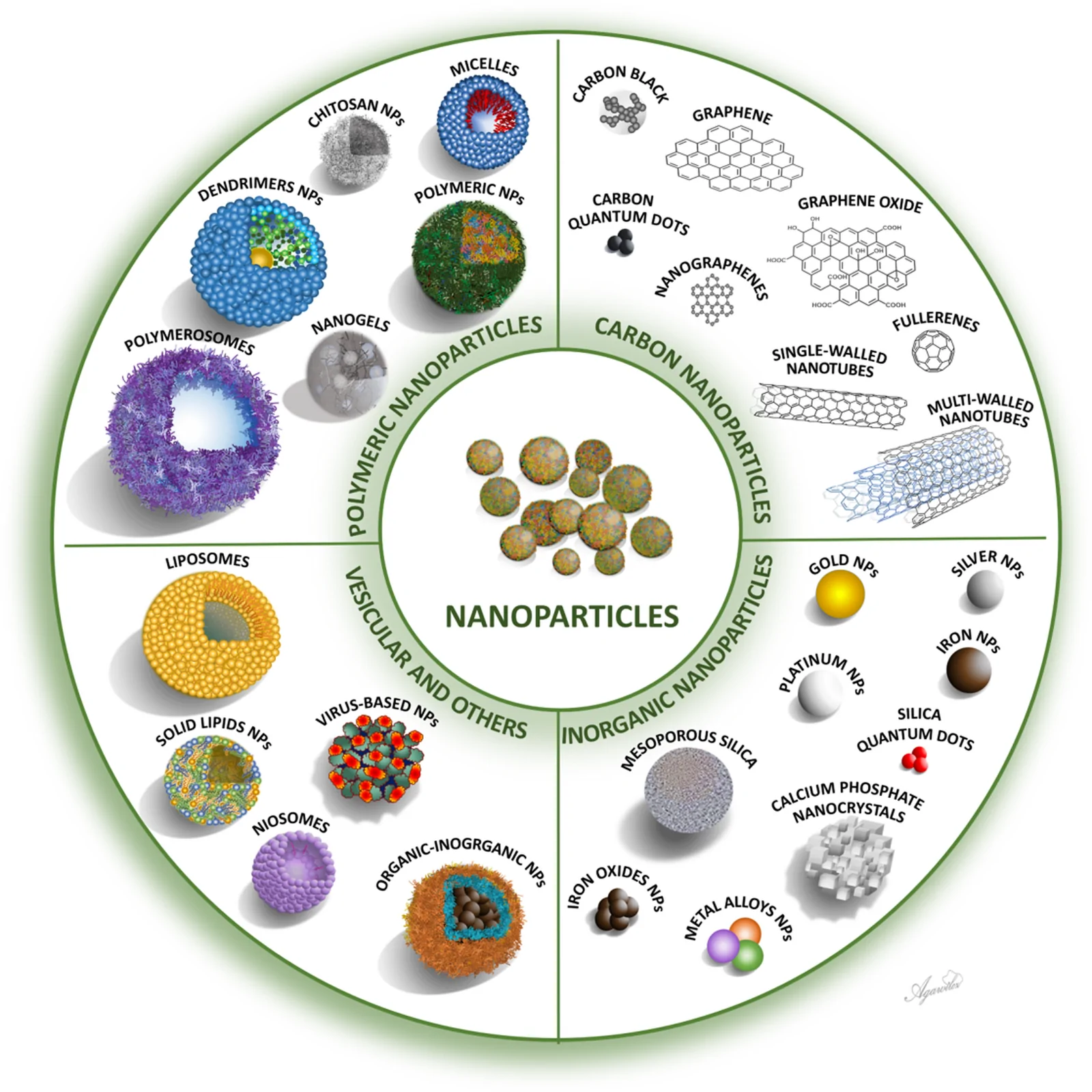
Types of nanoparticles used as nanocarriers according to their chemical composition. This figure summarizes the major classes of nanoparticles used as drug delivery systems, grouped by chemical composition and structural organization. The schematic includes polymeric, carbon-based, inorganic, vesicular, and hybrid nanocarriers, highlighting their structural diversity and multifunctional properties relevant to nanomedicine applications

Various nanoparticle-based drug delivery systems, including polymeric, carbon, inorganic, and vesicular NPs (Fig. 1), have been extensively investigated for biomedical applications. These systems exhibit diverse properties that enable the effective transport of active agents, targeted therapy, and biomedical imaging, integrating diagnostic and therapeutic functions within a single platform. However, it is important to note the significant limitations highlighted, including potential toxicity, system stability, and the complexity of nanoparticle synthesis and surface functionalization [37, 38].

## Biomedical applications of nanoparticles

### Cancer therapy

In contemporary cancer therapy, nanoparticle technology plays a prominent role, with applications in both therapeutic agent delivery and treatment outcome enhancement. Two targeting mechanisms are critical: passive and active. Passive targeting is predicated on the EPR (Enhanced Permeability and Retention) effect, which facilitates the selective accumulation of nanoparticles within tumor tissue. This phenomenon can be attributed to increased vascular permeability in the tumor and limited lymphatic drainage, resulting in local accumulation of nanocarriers and increased drug concentration at the lesion site, while concomitantly reducing systemic toxicity [39, 40]. Conversely, active targeting involves functionalizing nanoparticle surfaces with ligands, such as antibodies, aptamers, or peptides, that recognize receptors overexpressed on cancer cells. This mechanism facilitates specific binding and internalization of nanoparticles via receptor-mediated endocytosis. Moreover, this approach has the potential to circumvent multidrug resistance by bypassing drug efflux mechanisms from the cell [41].

The use of nanoparticles in cancer immunotherapy is also significant, as they serve as carriers for immunomodulators, antigens, and immune checkpoint inhibitors. These substances are then delivered effectively to the tumor microenvironment and antigen-presenting cells, promoting T-cell activation and enhancing the efficacy of the immune response [42]. In nanochemotherapy, anticancer drugs are encapsulated in nanocarriers such as liposomes, polymeric micelles, or gold nanoparticles. This approach has been demonstrated to enhance the pharmacological stability of the drugs in question and their transport within the body. Furthermore, these systems can utilize the EPR effect, which refers to the mechanisms of active recognition of cancer cells, and respond to specific conditions of the tumor microenvironment, such as pH, temperature, or redox conditions. This, in turn, increases the selectivity of the therapy [43].

### Diagnostics and imaging

The role of nanoparticles in modern diagnostic methods, particularly in imaging and biosensors, is significant. In magnetic resonance imaging (MRI), superparamagnetic iron oxide nanoparticles (Fe₃O₄, γ-Fe₂O₃) are utilized, thereby reducing the T2 relaxation time of water protons. This results in reduced signal intensity on T2-weighted images, thereby enabling improved visualization of pathological lesions as negative contrast. The effectiveness of these nanoparticles depends on their size, magnetic properties, and surface modifications, which influence colloidal stability and circulation time in the body [44]. Electrochemical biosensors are also employed in diagnostics, where the use of nanoparticles increases the electrode active surface area and enhances electron transport. This has been shown to increase sensitivity and detection speed for biomarkers such as glucose or tumor antigens. Additionally, nanostructures, such as carbon nanotubes and metal nanoparticles, facilitate device miniaturization and the development of multiparameter diagnostic systems [45]. A significant group consists of luminescent nanoparticles, which, thanks to their photoluminescent properties, enable sensitive biological analysis and imaging. These sensors are distinguished by their high photochemical stability, high signal intensity, and multiplexing capability, which enables the concurrent detection of multiple analytes. Quantum dots are of particular importance because their emission depends on size, enabling precise diagnostic applications [46].

### Antibacterial and antifungal applications

Metal nanoparticles have been demonstrated to exhibit antifungal activity against numerous pathogens, including *Candida albicans*, *Aspergillus spp*., and *Fusarium spp*. The mechanism of action of these compounds involves the generation of reactive oxygen species (ROS), damage to the cell membrane, and disruption of fungal cell metabolic processes. Furthermore, the use of nanoparticles has been shown to inhibit resistance mechanisms, including efflux pumps, and to impede biofilms formation, thereby enhancing their therapeutic efficacy [47]. Silver nanoparticles have been shown to exhibit similar properties; they interact with the microbial cell membrane, leading to its damage and, subsequently, disturbances in intracellular metabolism. A significant mechanism of their action is the induction of oxidative stress via ROS generation, leading to cell death [48].

### FDA-approved nanotherapeutics

The development of nanomedicine, coupled with the approval of the first nanotherapeutics by the U.S. Food and Drug Administration (FDA), signified a pivotal transition in the translation of nanotechnology from the laboratory to the clinical realm. The development of nanomedicine can be categorised into three distinct phases. The initial phase encompassed fundamental research conducted from the discovery of the structure of liposomes in 1964 until the clinical implementation of the first therapeutic nanosystems. The second stage, spanning from 1995 to 2007, was predominantly characterised by the clinical validation and commercialisation of nanotherapeutics. The third stage involves the dynamic development of modern therapeutic nanosystems and the introduction of further innovative nanomedicines into clinical practice. At present, more than 90 nanoscale drug delivery systems have been approved for commercial distribution [49].

A seminal moment in the evolution of nanomedicine was the FDA’s approval in 1995 of Doxil^®^/Caelyx^®^, a PEGylated liposomal formulation of doxorubicin. The utilisation of PEGylated liposomes facilitated an augmentation of the drug’s circulation time in the bloodstream, concomitant with a diminution in its uptake by the reticuloendothelial system. In addition, the drug exhibited reduced cardiotoxicity compared with free doxorubicin, and increased accumulation in tumor tissue as a consequence of the effect of increased permeability and retention (EPR) [50, 51]. Another approved pharmaceutical agent is DaunoXome^®^, a liposomal formulation of daunorubicin that was approved by the FDA in 1996 for the treatment of AIDS-related Kaposi’s sarcoma. The pharmaceutical compound was encapsulated within small, single-layer liposomes composed of phosphatidylcholine and cholesterol, resulting in an enhanced pharmacokinetic profile and increased drug accumulation in tumor tissue. In comparison with conventional daunorubicin, the formulation demonstrated reduced systemic toxicity and a more favourable safety profile [52, 53]. In 1999, the FDA approved the use of DepoCyt^®^, a pharmaceutical compound, for the treatment of lymphomatous meningitis. The formulation utilises DepoFoam™ technology, based on multivesicular liposomes that enable the slow, controlled release of cytarabine into the cerebrospinal fluid. This development enabled a substantial prolongation of the drug’s duration of action, and a reduction in the frequency of intrathecal administrations [54]. Conversely, Myocet^®^ is a non-PEGylated liposomal formulation of doxorubicin that was approved by the European Medicines Agency (EMA) in 2000 for the treatment of metastatic breast cancer. In contrast to Doxil^®^, the preparation lacks a PEG shell, yet it exhibits reduced cardiotoxicity compared with free doxorubicin. The liposomal encapsulation of the active substance has been shown to facilitate more selective drug distribution to tumor tissues, thereby reducing adverse effects [54].

The accelerated advancement of medical nanotechnology has also given rise to the development of lipid nanoparticles, which are utilised as mRNA delivery systems in the context of vaccines for the treatment of SARS-CoV-2, further underscoring the significance of nanomedicine in contemporary infectious disease treatment and prevention strategies [49]. The current clinical approval of nanotherapeutics encompasses both lipid- and polymer-based drug delivery systems, which are applied across a range of contexts, including oncology, infection treatment, hematological diseases, and modern biological therapies. The following section presents a selection of approved nanomedicines (Table 1).

**Table 1:** Examples of clinically approved nanomedicines introduced over the last two decades

Product Name	Active Drug	Nanoparticles Type	Main Indication	Approval Agency, Year	Key Advantage of Liposomal Formulation	Ref.
**Lipid -based nanoparticles**						
Mepact^®^	Mifamurtide	Multilamellar liposome	Osteosarcoma	EMA, 2009	Enhanced macrophage activation and immune stimulation	[55]
Marqibo^®^	Vincristine	Sphingomyelin/cholesterol liposome	Acute lymphoblastic leukemia	FDA, 2012	Improved pharmacokinetics and reduced neurotoxicity	[56]
Onivyde^®^	Irinotecan	PEGylated liposome	Metastatic pancreatic cancer	FDA, 2015	Increased tumor accumulation and prolonged circulation	[57]
Vyxeos^®^	Cytarabine + Daunorubicin	Dual-drug liposome	Acute myeloid leukemia	FDA, 2017	Fixed synergistic drug ratio and enhanced efficacy	[58]
AmBisome^®^	Amphotericin B	Conventional liposome	Fungal infections	FDA, 1997	Reduced nephrotoxicity	[59]
Arikayce^®^	Amikacin	Liposomal inhalation suspension	Mycobacterium avium complex lung disease	FDA, 2018	Improved pulmonary delivery	[60]
Comirnaty^®^	BNT162b2 mRNA	Lipid nanoparticle (LNP)	COVID-19	FDA/EMA, 2020	Efficient mRNA protection and intracellular delivery	[61]
Mosquirix^®^ vaccine	Recombinant CSP	Liposome-based adjuvant	Malaria	WHO 2021		[62]
**Polymeric Nanoparticles**						
Abraxane^®^	paclitaxel	Albumin based	pancreatic cancer and MBC	FDA 2005	Improved pharmacokinetic and tumor distribution	[63]
Genexol^®^ PM	paclitaxel	mPEG and poly-D, L-lactide (PDLLA)	MBC, NSCLC, and ovarian cancer	MFDS, PDH, CDSCO, DAV 2007	Enhanced tumor accumulation good biocompatibility and degradability	[64]
Mircera^®^	Methoxy polyethylene glycol-epoetin beta	Polymer-protein conjugate	CKD associated anemia	FDA 2007	Improved pharmacokinetic, long half-life	[65]
Rebinyn/Refixia	Coagulation Factor IX (Recombinant)	Polymeric nanoparticles GlycoPEGylated	Hemophilia B	FDA 2017	Improved pharmacokinetic, long half-life	[66]
Viva-Gel^®^	SPL7013	dendrimer with polyanionic surface,	preventing HIV and HSV viruses	FDA 2006	Inhibition 70% of HIV-1 and HSV-2, good safety and tolerance	[67]

CDSCO – Central Drugs Standard Control Organization (India); CKD – Chronic Kidney Disease; DAV – Drug Administration of Vietnam; EMA – European Medicines Agency; FDA – Food and Drug Administration; MFDS – Ministry of Food and Drug Safety (South Korea); MBC – Metastatic Breast Cancer; NSCLC – Non-Small Cell Lung Cancer; PDH – Pakistan Drug Authority / Health division (uncertain abbreviation); WHO – World Health Organization

Summarized lipid-based and polymeric nanocarriers have significantly improved the therapeutic efficacy and safety profiles of numerous drugs. Liposomal formulations, including PEGylated, conventional, and multivesicular liposomes, as well as polymer-based nanostructures, have demonstrated major advantages. The application of nanoformulation improves pharmacokinetics, enhances biocompatibility, prolongs the half-life of therapeutic agents, and improves their biodistribution. As a result, they can achieve enhanced tumor accumulation, sustained drug release, and reduced systemic toxicity, particularly cardiotoxicity and nephrotoxicity. These systems have been successfully applied in oncology, infectious disease treatment, and vaccine development, emphasizing their broad clinical applicability

## Types of nanoparticles used as drug carriers

Research on drug carriers has been ongoing for many years. The variety of chemical compounds used for their creation is very large and continues to grow. Nanoparticles can vary in chemical composition, shape, size, and surface properties. Their morphology ranges from simple spherical particles to more complex geometries, including rods, cubes, stars, hexagons, and core–shell architectures. The surface charge of nanoparticles is commonly expressed as zeta potential. It strongly affects their colloidal stability and interactions with biological systems. Positively charged NPs often support cellular uptake, though they may also increase toxicity. On the other hand, neutral or negatively charged nanoparticles are generally more biocompatible.

Sizes typically range from 1 to 100 nm. According to the International Organization for Standardization, nanoparticles are defined as particles with diameters between 1 and 100 nm [68]. The American Society for Testing and Materials also applies a 1–100 nm range, typically in two or three dimensions [69]. The International Union of Pure and Applied Chemistry uses the same dimension range, 1–100 nm, as the requirements for NPs. IUPAC, however, extends this limit to 500 nm [70]. The Scientific Committee on Emerging and Newly Identified Health Risks refers to nanomaterials as those in the 1–100 nm range [71]. The European Commission Recommendation 2011/696/EU states that if at least half of the material’ particles (by number) have one or more external dimensions between 1 and 100 nm, they can be considered as a nanomaterial [72].

Their chemical composition may vary widely. Nanocarriers can be divided into four main groups: (1) polymeric NPs (e.g., micelles, dendrimers, nanogels), (2) carbon NPs (e.g., carbon black (CB), single- and multi-walled nanotubes (SWNTs, MWNTs), graphene, graphene oxides, fullerenes), (3) inorganic NPs (e.g., metals, alloys, ceramics, silica), (4) vesicular and other NPs (liposomes, solid lipid NPs, virus-based NPs, organic-inorganic hybrid) (see Fig. 1). Nanocarriers can be composed of one or more different components. Among multicomponent systems, known polymer-lipid or polymer-inorganic hybrids, as well as conjugates that incorporate therapeutic agents, imaging probes, or targeting ligands, exist. This diversity in structure and composition makes NPs multifaceted platforms for nanomedicine applications. At the same time, it also calls for a comprehensive evaluation of the safety and functionality of those nanocarriers.

## Cellular effects of nanoparticles

**Fig. 2:**
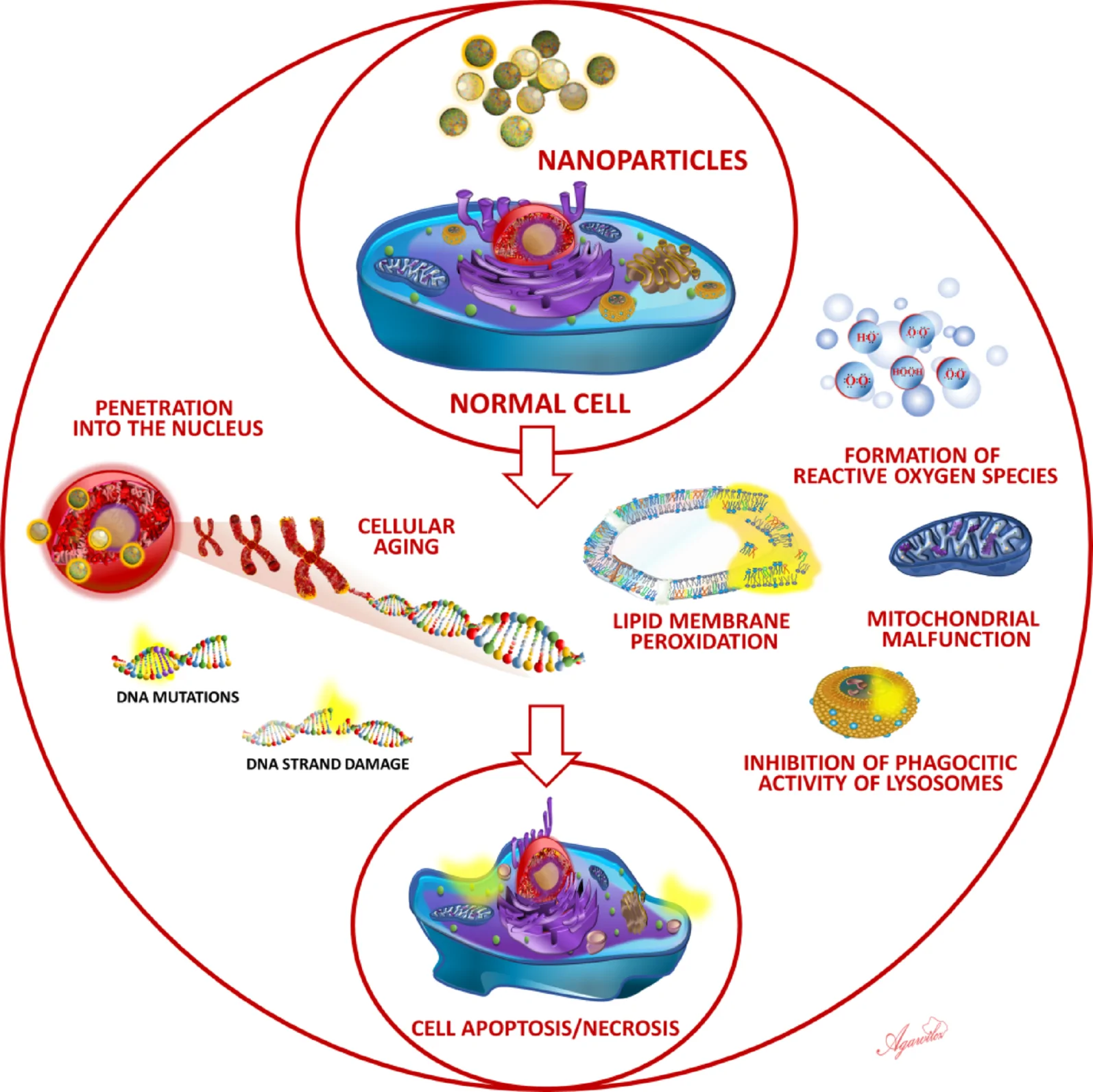
Cellular effects of nanoparticles. Summary of the major cellular and molecular mechanisms involved in nanoparticle-induced toxicity. Following cellular internalization, nanoparticles may trigger ROS generation, lipid peroxidation, mitochondrial dysfunction, lysosomal impairment, DNA damage, chromosomal instability, and cellular senescence. These processes may subsequently induce apoptosis, necrosis, and inflammatory responses, thereby contributing to nanoparticle-associated cytotoxicity and tissue injury

The main cellular effects are presented in Fig. 2. The drug delivery system undoubtedly has many advantages that far outweigh its side effects. However, it should be emphasized that they may appear not only from the medicinal substances but also from the carriers themselves. Negative effects can occur both at the target site and in adjacent cells, tissues, and other organs. Some of the carriers (e.g., polymeric nanocarriers polyethylenimine (PEI) and poly(2-dimethylaminoethyl methacrylate) (PDMAEMA)) are not biodegradable in the body, are characterized by high cytotoxicity, and generate reactive oxygen species (ROS) (Fig. 2) [73, 74]. This is particularly detrimental when the same drug is administered repeatedly [73–75]. ROS can also be generated by silver nanoparticles (Ag NPs). The authors demonstrated that Ag NPs can induce ROS production, leading to endothelial cell damage via mitochondrial dysfunction [76]. A similar effect has been reported for copper oxide nanoparticles (CuO NPs), zinc oxide nanoparticles (ZnO NPs), gold nanoparticles (Au NPs), cerium oxide nanoparticles (CeO_2_ NPs), and iron oxide nanoparticles (Fe_2_O_3_ NPs) [77]. Although ROS is naturally and constantly present in the human body, its production can increase significantly in the presence of a physiological imbalance. The consequence of this phenomenon is the destruction of cellular components, particularly the cell membrane, which is integral to cellular structure and function. When its continuity is disrupted, cellular elements spill out, leading to extensive inflammation. Excessive production of ROS and their side effects have been studied and described by many authors. The negative effects of their action at the cellular level may include enzyme inactivation by damaging key amino acids (e.g., cytosine, tyrosine, threonine, and serine), peroxidation of membrane lipids (Fig. 2), structural changes in proteins and carbohydrates, and DNA damage. ROS can also alter the activity of nonenzymatic proteins [78], leading to frequent loss of their biological function. This harms the metabolism of nerve cells. In the nervous system, such phenomena lead to disturbances in neuronal homeostasis, often resulting in neuronal death via necrosis or apoptosis [79, 80]. Also, nucleic acids and proteins may be compromised by excessive ROS production, contributing to cellular dysfunction in multiple tissues and organ systems. Oxidative stress has been implicated in mechanisms associated with neurodegenerative disorders such as Parkinson’s disease and Alzheimer’s disease, as well as ocular, cardiovascular, and respiratory diseases. In severe cases, ROS-induced DNA damage (Fig. 2) may additionally promote mutagenesis and cancer development [81–83]. Studies conducted to date may indicate potential side effects associated with using nanoparticles of different shapes, including CB, short carbon nanotubes, long and entangled carbon nanotubes, and long, needle-shaped carbon nanotubes (CNTs) as drug carriers. Based on many analyses, numerous neurodegenerative diseases can be triggered by ROS; therefore, the use of CNTs as drug carriers may cause similar side effects due to their increased production. In addition, indirect negative effects of drug carriers on DNA, through modifications, deletions, and base additions, can cause reading frame changes, increased expression of protooncogenes, and other anomalies, leading to cell death [84].

Despite their numerous benefits, the use of nanoparticles as drug carriers poses a risk to cells due to their nanoscale dimensions. Nanocarriers with a size of 8–10 nm can penetrate the cell nucleus through its pores, whereas larger particles (15–60 nm) enter the nucleus when it divides, even causing direct DNA damage. Indirect effects are also dangerous because the large number of molecules involved in gene function can carry nanoparticles into the nucleus [4, 85, 86]. Changes or damage in the DNA structure can lead to three main effects: aging of the cell through irreversible loss of cellular activity, apoptosis of the cell, or the appearance of mutations. Mutations are the substrate for tumorigenesis by inducing uncontrolled cell division. In addition, if ROS are generated, they react with nitrogenous bases in DNA, leading to damage or the formation of highly mutagenic molecules such as 8-oxoguanine. That, if not corrected by repair processes, promotes tumor formation [87]. Changes in cell morphology caused by excessive ROS, responsible for oxidative stress, lipid peroxidation, and mitochondrial dysfunction, can be induced by even small amounts of carriers, such as single-walled carbon nanotubes (SWCNTs) [4, 88].

Wan B et al. reported that acid-functionalized single-walled carbon nanotubes (af-SWCNTs) and graphene oxides exhibit concentration-dependent cytotoxicity in cells. They also investigated the impact of MWNT on lysosomal function (Fig. 2), finding that abnormal autophagy process leads to malfunction of these cellular organelles [89]. On the other hand, Dong et al. studied the accumulation site of SWCNTs, which was found to be lysosomes, where nanotubes are taken up by macrophages, thereby inhibiting phagocytic activity and impairing. Decreased phagocytic activity directly correlates with less effective eradication of microorganisms, which is detrimental to the body due to a higher risk of disease, depending on the pathogen that remains uneradicated by phagocytosis [90].

**Fig. 3:**
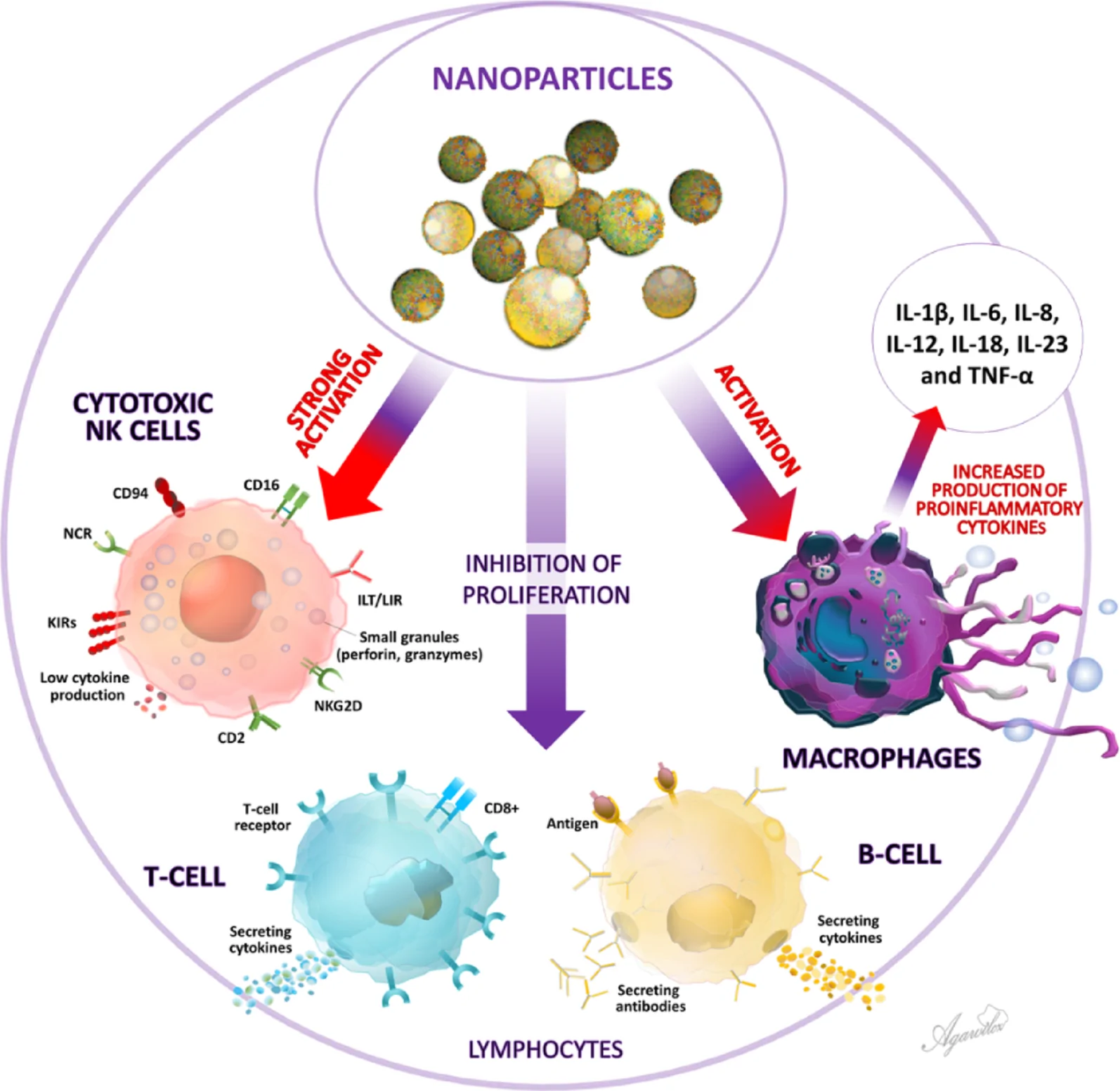
Effects of nanoparticles on immune cell function. This schematic illustration presents the immunomodulatory and immunotoxic effects associated with nanoparticle exposure. Nanoparticles may induce strong activation of macrophages and cytotoxic NK cells, accompanied by enhanced secretion of proinflammatory cytokines, including IL-1β, IL-6, IL-8, IL-12, IL-18, IL-23, and TNF-α. At the same time, nanoparticles may suppress lymphocyte proliferation and impair the activity of T and B cells, thereby contributing to immune imbalance, persistent inflammation, and dysregulation of immune homeostasis

On the other hand, Li Y. et al. have studied how SWCNTs affect macrophages. Their work showed that regardless of concentration, nanocarriers affect the initiation of necrosis and apoptosis in macrophages (Fig. 3) [91]. Furthermore, multi-walled carbon nanotubes (MWCNTs) have been shown to exacerbate oxidative stress in macrophages of various lineages by promoting cellular ROS production [92]. The term “oxidative stress” refers to an imbalance between ROS production and the cell’s ability to neutralize them. The primary ROS are the superoxide anion radical (O₂•⁻), hydrogen peroxide (H₂O₂), and the hydroxyl radical (•OH). The primary source within the cell is the mitochondrial electron transport chain, where electron “leakage” occurs, leading to the premature reduction of molecular oxygen. Under physiological conditions, a small amount of ROS performs signaling functions; however, an excess of ROS, particularly during aging, inflammation, or intense metabolism, leads to damage to cellular structures. O_2_^•−^ undergoes dismutation to H_2_O_2_, which can be further converted to the highly reactive •OH or react with nitric oxide to form nitrosyl peroxide (ONOO-), which is highly toxic to cells. The body counteracts these processes through an antioxidant system comprising enzymes such as superoxide dismutase (SOD), catalase (CAT), glutathione peroxidase (GSH-Px), and peroxiredoxins. SOD catalyzes the conversion of O_2_^•−^ to H_2_O_2_, while CAT and GSH-Px reduce H_2_O_2_ to water, thereby preventing the formation of the most reactive hydroxyl radical. Concurrently, non-enzymatic antioxidants, including glutathione, vitamins C and E, carotenoids, alpha-lipoic acid, and melatonin, are engaged in neutralizing free radicals or augmenting the enzymatic system. Trace elements (e.g. zinc, copper, selenium), which act as cofactors for antioxidant enzymes, also play an important role [93]. Enhanced oxidative stress in cells, induced by an excess of free radicals and superoxides, leads to a range of adverse biological effects. Among them is damage to cellular components, including the most sensitive proteins and DNA. As a consequence, necrosis can occur when ATP levels are reduced, preventing the cell from entering the apoptotic pathway [94, 95]. Oxidative stress can also be a pathogenic factor. It can lead to neurodegenerative diseases through neuronal damage [96], cardiovascular diseases, in which free radicals are important in the development of hypertension. This leads to consequences such as atherosclerosis, ischemic heart disease, stroke, or even myocardial infarction. The use of nanocarriers for drug delivery may also be unfavorable in the case of cancer, since oxidative stress, in addition to inducing apoptosis of cancer cells, may initiate tumor progression [97]. Additionally, it may lead to the formation of subsequent mutations [97], promote tumor cell migration leading to metastasis and stimulate angiogenesis promoting tumor growth [96].

Yang et al. and Tahara et al. studied the effects of SWCNTs and MWCNTs on macrophages. In both cases, the researchers observed lysosomal membrane damage due to nanoparticle accumulation, leading to excessive ROS production, apoptosis, and necrosis in macrophages (Fig. 2) [98]. Furthermore, pristine graphene induces apoptosis in macrophages [91]. Park et al. studied the effects of taurine-functionalized multi-walled carbon nanotubes (tau-MWCNTs), acidic MWCNTs, and SWCNTs on RAW264.7 macrophages. They found out that nanoparticles caused a dose-dependent decrease in ATP production, mitochondrial dysfunction, and increased ROS production, leading to apoptosis in treated cells [99]. It should be emphasized that impaired mitochondrial function can disrupt cellular calcium homeostasis [100]. Mitochondrial dysfunction is particularly detrimental to tissues with high energy demands, such as the nervous system, heart, and skeletal muscle. For instance, the brain alone consumes up to 70% of the body’s ATP, and mitochondrial dysfunction is a known contributor to neurodegenerative diseases. Similarly, in the heart and muscle tissue, high metabolic sensitivity means that mitochondrial impairment can manifest as sudden changes in heart rhythm and attacks of unexplained fatigue. Additionally, decreased ATP production can disrupt the synthesis of neurotransmitters like dopamine and serotonin, leading to an increased tendency for depression [100].

The novel and related effects of CB NPs on the macrophages were identified by Ritters et al. Authors reported that they induce not only apoptosis and necrosis but also pyroptosis, an inflammatory type of programmed cell death [101]. This process was characterized by pore formation in the cell membrane, disrupting its continuity and consequently leading to a loss of cellular homeostasis [102]. The researchers further linked these effects to inflammasome activation and the release of the cytokine IL-1β [101].

## Immunological effects of nanoparticles

The role of NPs in the immune system is multifaceted. These particles have been observed to engage with the immune system, thereby modulating its function. The consequences of this engagement are twofold, as it can result in either immunosuppression or immunostimulation. The effects on the immune system may promote the treatment of diseases by potentiating immune responses. Conversely, the immunomodulatory effects of NPs can be sufficiently potent to induce adverse effects. The physicochemical properties of nanoparticles are primarily responsible for their adverse immunotoxic effects. Besides structure, composition, surface functionalization, and protein-binding ability, the route of administration is also critical, as it can lead to different side effects, ranging from acute inflammation to systemic damage [103–105]. A primary immunotoxic effect of drug carriers is their negative impact on the immune system. Table 2 summarizes examples of research on the immunomodulatory effects of NPs used in nanomedicine.

Researchers frequently report that the immunotoxic effects of NPs are manifested as increased production of proinflammatory cytokines and elevated ROS levels. Additionally, ATP deficiency is known to negatively affect the immune system by promoting excessive production of pro-inflammatory cytokines and free radicals, ultimately reducing immune function [106]. For instance, Hamilton et al. and Zang et al. reported a relationship between the concentration of administered MWCNTs, including those PEG- and COOH-functionalized, and the enhanced production of TNF-α, IL-1β, and IL-6 (Fig. 3) [107, 108]. Additionally, Delogu et al. investigated the effect of functionalized CNTs on natural killer (NK) cells and B and T lymphocytes, finding that the nanocarriers strongly activated NK cells [109].

The study also analyzed the effect of nanoparticle shape, including CB NPs and tangled, short, long, or needle-like CNTs, on immunotoxicity. Exposure to these variably shaped nanoparticles consistently led to increased cytokine production, particularly from the IL-1 family (Fig. 3). This IL-1 response has significant pathological consequences. IL-1 family cytokines, especially IL-1β, are potent pyrogens (fever-inducing) and central regulators of immunity, activating dendritic cells and driving B- and T-cell responses [110]. These processes can manifest clinically as rashes and, in severe cases, arthritis. Crucially, the activation of B lymphocytes is a key link in the pathogenesis of autoimmune disease. B cells produce antibodies, including IgM autoantibodies that target host antigens and are recognized as a factor in autoimmune disease development [111, 112]. This is highly relevant, as B-cell proliferation has been documented in several inflammatory lung diseases, including chronic obstructive pulmonary disease [113, 114], bronchial asthma [114], and during disease progression [115]. A significant increase in bronchial B lymphocytes was also found in individuals who died from an acute asthma attack [116]. The former reports indicate that nanoparticle exposure can drive harmful immunological changes, particularly inappropriate B-cell activation, that mirror those seen in serious autoimmune and inflammatory diseases. The mechanism underlying this IL-1-driven inflammation involves a well-defined cellular pathway. For instance, Palomaki et al. reported that CNTs promoted ROS production and cathepsin B activation, which are essential for the production of tumor necrosis factor (TNF-α) in mouse macrophages [117]. As detailed by Ha et al., the pathway engagement of cathepsin B and ROS subsequently activates the NOD-like receptor family, pyrin domain-containing 3 (NLRP3) inflammasome, a complex that stimulates IL-1ß and IL-1 secretion from human monocyte-derived macrophages (HMDMs). As described above, the release of these cytokines can trigger systemic inflammatory symptoms such as fever, rash, and arthritis [118].

Other researchers have focused more on the concentration of a given carrier than on the type itself. Sun et al. found that low doses of CNTs (0.00–0.1 µg/mL) are not toxic to human lymphocytes. However, upon activation of the nuclear factor (NF-κB), CNTs increased cytotoxicity [119]. In contrast, Zhang et al. observed that higher concentrations of SWCNTs inhibited the proliferation of T lymphocytes. In case of B lymphocytes, all tested concentrations (1, 10, 25, and 50 µg/mL) inhibited proliferation [120]. Also, natural killer (NK) cell activity was significantly reduced. The same study also examined the effect of SWCNTs on spleen cells and found that lower concentrations had little impact, whereas higher concentrations increased proliferation [88]. In turn, Reisetter et al. observed inflammasome activation and IL-1β release in response to CB NPs. Stimulation of this interleukin can lead to activation, the synthesis of acute-phase proteins, and the induction of other cytokines, such as IFN-γ, IL-6, and TNF (Fig. 3). The resulting systemic effects include lethargy and potential fever, as IL-1β is a pyrogen [101]. Additionally, IL-1β can affect cellular insulin sensitivity [121, 122]. More broadly, activation of the inflammasome leads to the secretion of proinflammatory interleukins (e.g., IL-1 and IL-18) and causes cellular damage and aberrant cell death via pyroptosis [101]. In conclusion, nanoparticles might stimulate the immune system, leading to excessive cytokine production [123]. This overproduction is a biomarker of immunotoxicity [124] and can often lead to hypersensitivity, inflammatory reactions, and in severe cases, even death [4, 125]. However, it is important to note that certain properties of the nanoparticles may also be employed in a range of promising applications. In these strategies, the immune response to nanoparticles can be utilized to achieve specific objectives, such as in vaccination and cancer immunotherapy [126, 127].

**Table 2:** Immunological properties of various classes of nanoparticles

Nanoparticles	Size [nm]	Physicochemical features	Model	Effect	Ref.
**Polymeric NPs**					
PAMAM 2nd and 3rd	2.9–3.6	ND	In vitro, HDFa and HEK cells	IL-8 ↑, IL-1β↑ IL-6↑	[128]
NACOS	ND	Water-soluble	In vitro RAW cells	↑ mRNA of TNF-α, IL-6, IL-1β	[129]
OS-PEG-Man	18.78 ± 3.07	Zeta potential H_2_O: +33.02 mV PBS: +4.21 mV	In vitro HL-60 and THP-1 cell lines	IL-8 ↑	[130]
**Carbon NPs**					
GNS	2	Zeta potential: − 33.17 ± 2.04 mV	In vivo C57BL/6 male mice	IL-5↑, IL-13↑, IL-33↑	[131]
MWCNTs	20–30	Zeta potential: -20.79 ± 1.52 mV	In vivo C57BL/6 male mice	IL-4↑, IL-33↑, CD4+↑, CD8+↑	[131]
	∼10–20	ND	In vivo C57BL/6 male mice	IL-10↑	[132]
	67	ND	In vivo male ICR mice, in vitro immature BMDC	IL-4↑, IL-5↑, IL-13↑, IFN-γ↑, IL-18↑, IL-33↑	[133]
CB	< 50	ND	In vitro human monocytic cell line THP-1	IL-1 β↑, IL-6↑, TNF- α↑, IL-8↑	[134]
**Inorganic NPs**					
ZnO NPs	32–40	ND	In vitro A549 cells In vivo male Sprague Dawley (SD) rats,	NLRP3 ↑, IL-1β↑,	[135]
Ag NPs	34–40	ND	In vitro, THP-1 cells	IL-1↑, IL-6↑, TNF-α↑	[136]
Si NPs	16.75 ± 3.38	Zeta potential in: H_2_O: -25.95 ± 0.63 mV, RPMI-1640 containing 10% FBS: -16.81 ± 0.5 mV, suggesting SiNPs aggregation	C57BL/6 mice	IL-1β↑, IL-6↑	[137]
Fe_3_O_4_ NPs	20	ND	ICR mice	IL-2↑, IFN-Ɣ↑, IL-10↑	[138]
PEG-coated Au NPs	13	ND	In vivo male BALB/c mice	IL-1β↑, IL-6↑, IL-10↑, IL-12β↑, TNF-α↑	[139]
SiO₂ NPs	30–70	Well-dispersed smooth-surfaced spheres	In vitro RAW264.7, in vivo female BALB/c mice	TNFα↑	[140]
CeO_2_ NPs	∼20	Tendency to aglomeration	In vivo male Sprague Dawley rats	IL-12↑	[141]
QDs	ND (concentration = 2 nmol/kg)	ND	In vivo BALB/c male mice, In vitro	IL-6↑, TNF-α↑	[142]
**Vesicular and others NPs**					
Ag NPs&PS NPs mixture	20	ND	Human macrophages	IL-6 ↑, IL-8↑ TNF-α↑	[143]

Ag NPs – Silver nanoparticles, Ag NPs & PS NPs mixture – Mixture of silver nanoparticles (Ag NPs) and polystyrene nanoparticles (PS NPs), CB – Carbon black, CeO₂ NPs – Cerium dioxide nanoparticles, Fe₃O₄ NPs – Iron(II, III) oxide nanoparticles/magnetite nanoparticles, GNS – Graphene nanosheets, IL – Interleukin, MWCNTs – Multi-walled carbon nanotubes, NACOS – N‑acetyl chitooligosaccharides, OS-PEG-Man – Maltose-decorated poly(propylene imine) (PPI) dendrimer (OS) modified with six poly(ethylene glycol) (PEG) linkers, and mannose moieties attached to the PEG chains, PAMAM 2nd – Polyamidoamine dendrimer, 2nd generation, PAMAM 3rd – Poly(amidoamine) dendrimer, 3rd generation, PEG-coated Au NPs – Poly(ethylene glycol)-coated gold nanoparticles, QDs – Quantum dots, Si NPs – Silica nanoparticles, SiO₂ NPs – Silica dioxide nanoparticles, TNF-α – Tumor necrosis factor alpha, ZnO NPs – Zinc oxide nanoparticles

Summary of the toxicological effects of selected nanoparticle-based drug delivery systems observed in in vitro and in vivo experimental models. The table presents a compendium of typical biological responses observed across various nanoparticle systems, including oxidative stress, inflammatory signalling, cellular dysfunction, changes in the microbiome, and organ-specific toxicity. ↑ increase/↓ decrease in activity, expression, accumulation, permeability, or biological response

## Animal studies on nanoparticles

**Fig. 4:**
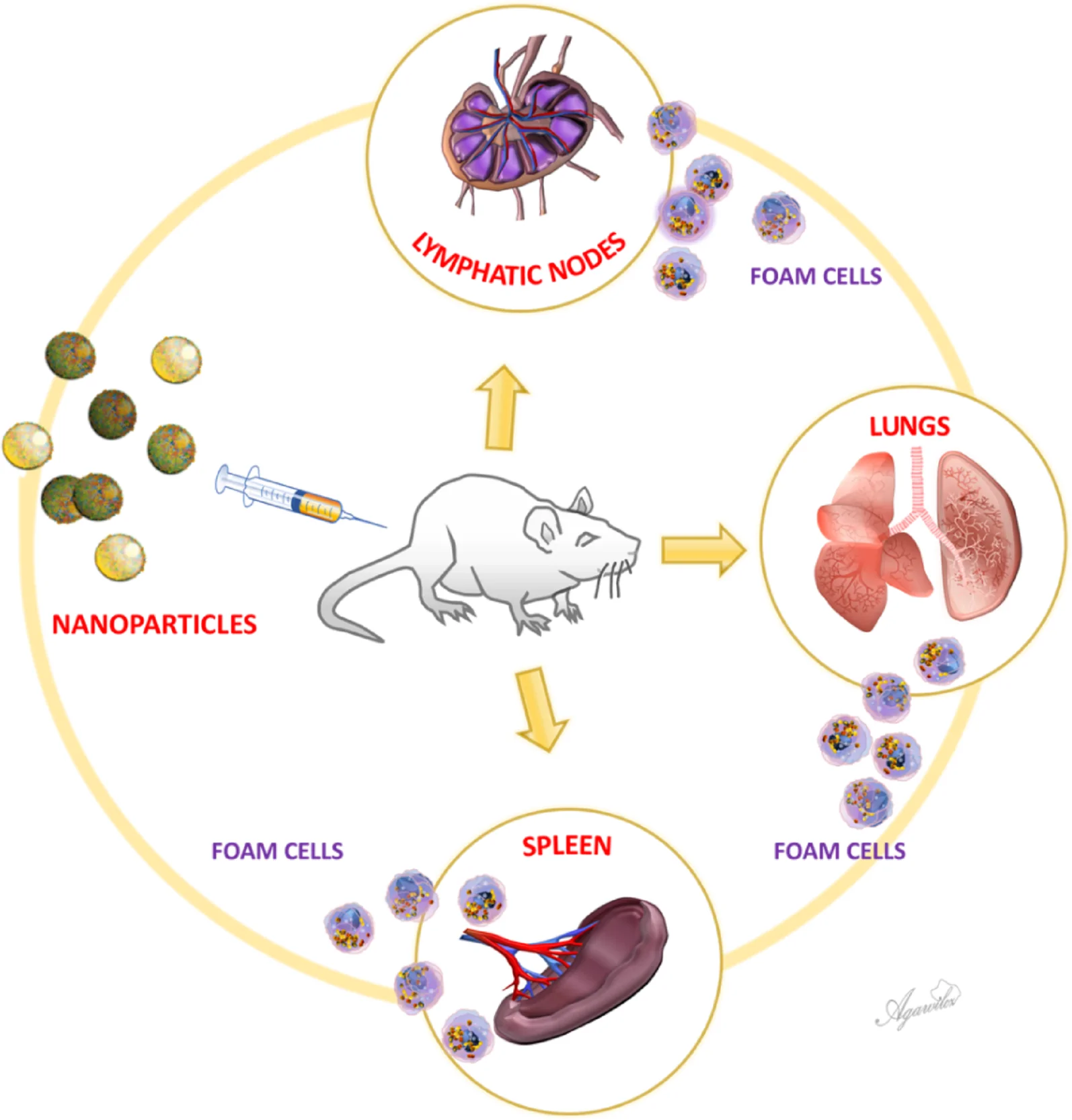
Biodistribution and cellular effects of polymeric nanoparticles in a rat model. This schematic illustration presents the biodistribution and selected biological effects of nanoparticles observed in experimental animal models. Particular emphasis is placed on nanoparticle accumulation in the lungs, liver, spleen, and lymph nodes, as well as on macrophage activation and foam cell formation. These alterations may contribute to chronic inflammation, impaired immune responses, tissue dysfunction, and metabolic disturbances associated with prolonged nanoparticle exposure

Shiraishi and Yokoyama described a study that investigated the toxicity of polymeric micelle nanoparticles in Donryu rats. The carriers were administered to the animals at either a low (20 mg/kg) or a high (200 mg/kg) dose, once every other day or daily, respectively. Rats were sacrificed after 8 and 30 days. Their major organs (i.e., heart, brain, liver, lungs, spleen, and kidneys) were examined histologically and immunohistochemically. No toxic effects of the polymeric micelles on these organs were observed. However, an increase in the number of foam cells in the lungs, lymph nodes, and spleen was observed in rats receiving the high dose of the carrier. The above has been confirmed by immunohistochemical examination, which revealed CD68-positive macrophages in the lungs, spleen, and liver (Fig. 4) [7]. Foam cells are a subset of macrophages that are considered targets in the treatment of infectious and metabolic diseases and are also implicated in certain cancers and autoimmune disorders [144–146]. These cells can also form due to lipid imbalance; excess lipids form droplets, giving the cells a characteristic foamy appearance. The presence of foam cells in the body is associated with various diseases, including atherosclerosis, multiple sclerosis, severe lung damage, certain cancers, and obesity. In atherosclerosis, for instance, these cells contribute to the formation and development of unstable atherosclerotic plaques [147]. Maddock et al. reported the presence of foam cells in the bronchoalveolar lavage of patients with severe lung injury [148]. Similarly, other authors have observed in central nervous system lesions in patients with multiple sclerosis [149]. A link to certain cancers has also been established, as foam cells have been shown to promote tumor initiation and progression [150]. Shapiro et al. and Coats et al. reported that foam cells are present in the adipose tissue of obese humans and in C57BL/6 mouse models. In a study on C57BL/6 mice, researchers demonstrated that the presence of these cells impaired adipose tissue response to insulin, thereby promoting obesity [151]. Furthermore, foam cells have been implicated in the development of chronic inflammation within adipose tissue [152]. Cellular effects of the rat model were presented in Fig. 4.

Wibroe et al. studied the effects of polystyrene nanoparticles on pigs. They reported that pulmonary intravascular macrophages (PIMs) were the most affected cell type due to rapid nanoparticle uptake, which led to a sudden surge in pulmonary artery pressure [153]. In a separate study, Tseng et al. used fluorescent gelatin nanoparticles as an inhaled drug carrier in both tumor-bearing and healthy mice models. In healthy animals, the nanoparticles were rapidly excreted by the kidneys, whereas in diseased mice they persisted in the lungs for 24 h. Furthermore, nanoparticles were detected in the tracheal epithelial cells as early as 30 min after administration [154]. Other studies have shown that gelatin nanoparticles can be located in the brain, heart, liver, spleen, and blood vessels [155]. In one such study, when doxorubicin was added to the nanoparticles, they accumulated exclusively in the lungs [155]. This targeted accumulation highlights that the long-term presence of nanoparticles in specific organs could be harmful.

The immunomodulatory effects of uncoated Au NPs have also been investigated in mice. Researchers demonstrated that these nanoparticles suppressed immune activity; administration at a concentration of 7.85 µg NPs/g reduced IL-6 and Toll-like receptor (TLR) expression in mouse adipose tissue [92]. Such suppression can inhibit cytokine activity, leading to a diminished or absent immune response. For example, the pyrogenic effect of IL-6 might be revoked, preventing fever in the presence of a pathogen. Similarly, T lymphocyte activation could be impaired, and the non-specific immune response typically mediated by TLRs may not be stimulated.

Beyond the side effects already described, animal studies have shown that nanoparticles can adversely affect reproductive organs by inducing cellular stress and inflammation [156]. For example, the accumulation of foam cells in adipose tissue can lead to chronic inflammation and insulin resistance [157].

## Preclinical evidence of nanoparticle toxicity relevant to human health

**Fig. 5:**
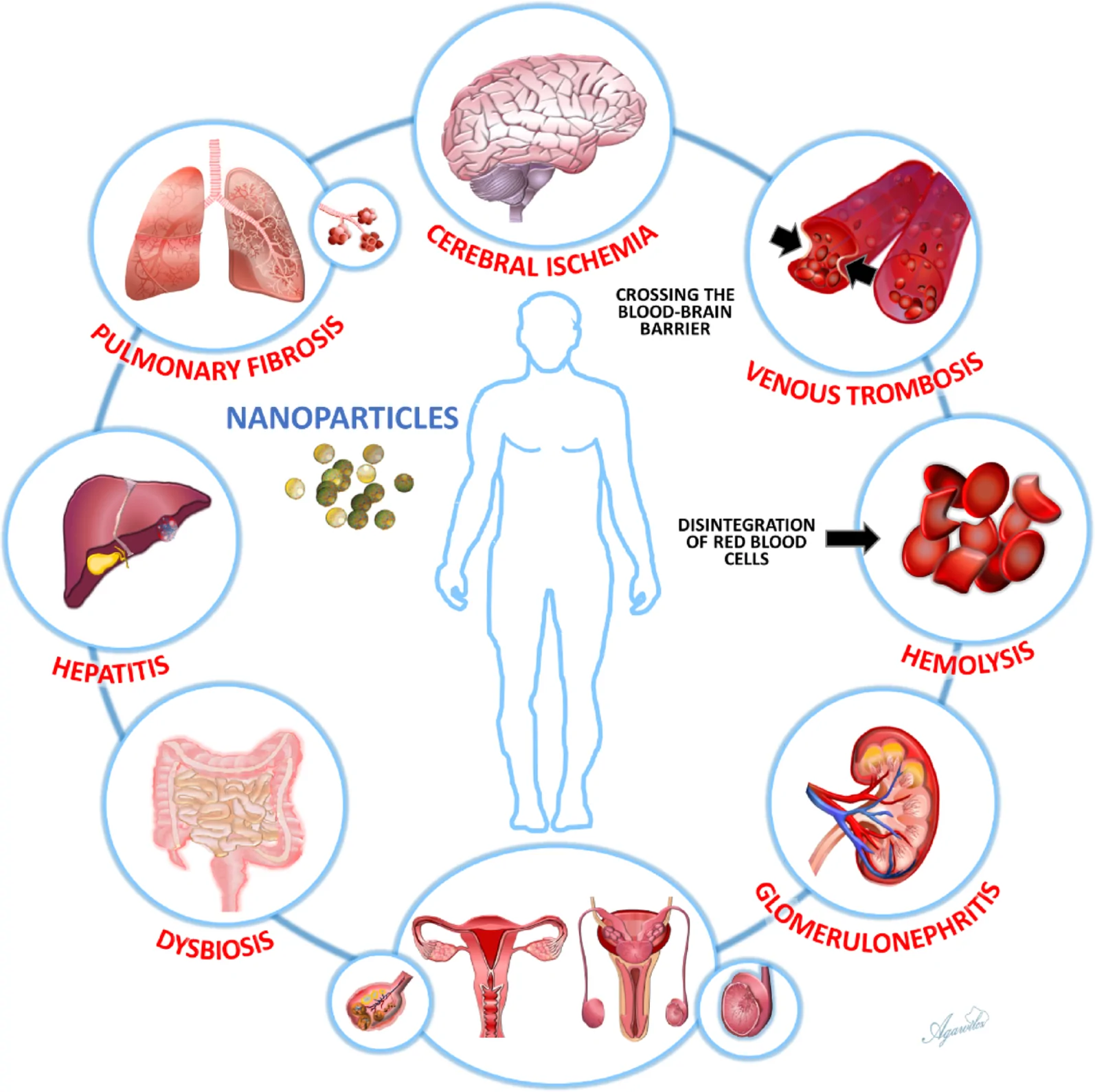
Predictable effects of exposure to nanoparticles on human organs and tissues. The figure illustrates the multisystem biological effects associated with nanoparticle exposure and accumulation in the human body. Following translocation across biological barriers, nanoparticles may induce pulmonary injury, cardiovascular disturbances, hepatotoxicity, nephrotoxicity, neurotoxicity, vascular dysfunction, and immune imbalance through mechanisms involving oxidative stress, inflammation, and cellular damage. The presented pathways emphasize the systemic character of nanoparticle-associated toxicity and the role of biodistribution in determining organ-specific adverse effects

Based on current research, it is well established that nearly every component of a drug carrier may induce adverse effects not only at the target site but also in non-target tissues and organs. Importantly, the biological response to nanocarriers is not determined solely by the core material, but is also strongly influenced by subsequent surface modifications, including coatings, functionalization, and the immobilization of therapeutic agents or targeting ligands. However, systematic comparative studies evaluating the toxicity and biodistribution of bare nanoparticles versus their surface-modified or drug-loaded counterparts remain limited. This lack of standardized head-to-head analyses hampers a comprehensive understanding of how specific structural modifications translate into changes in safety and biological behavior. Consequently, current interpretations of toxicity data are often based on heterogeneous experimental systems, which complicate direct comparisons and limit the predictability of clinical outcomes. The carrier’s presence can alter all the drug’s pharmacokinetic parameters, particularly its metabolism and excretion. Furthermore, nanoparticles can interact directly with cells and biological systems, leading to unintended side effects. These unwanted interactions most frequently affect the target tissue, organs responsible for detoxification (such as the liver, kidneys, spleen, and lymphatic system), blood components, vessel walls (Fig. 5), and the immune system [75]. However, extrapolating findings from in vitro and animal studies to humans remains limited due to differences in nanoparticle biodistribution, metabolism, immune responses, and long-term safety profiles between experimental models and clinical conditions. Therefore, in this chapter, based on available in vitro and in vivo studies, we discuss the potential consequences and possible human implications associated with the use of nanoparticle-based carriers.

Parhiz et al. provide a comprehensive overview of the effects of drug carriers, particularly nanoparticles, on biological systems, including major organs, tissues, blood components, and the immune system [75]. The lungs are considered the organs most susceptible to nanoparticle toxicity, particularly following inhalation exposure. Accumulation of nanoparticles in the pulmonary tissue may lead to edema, pulmonary hypertension, chronic inflammation, and fibrosis (Fig. 5). In an in-depth review, Zhou et al. provide a comprehensive summary of the findings, which indicate that carbon-based nanoparticles, metal-based nanoparticles, oxide-based nanoparticles, and sulfide-based nanoparticles induce pulmonary inflammatory responses in mice or rats after respiratory exposure [158]. Similarly, exposure to CuO NPs has been associated with edema and chronic inflammation, which may progress to pulmonary fibrosis [159]. From a clinical perspective, persistent pulmonary inflammation and fibrosis may impair respiratory function and potentially contribute to chronic respiratory diseases. Nevertheless, it should be emphasized that many of these observations were obtained under experimental exposure conditions that may not accurately reflect realistic human exposure levels or administration routes used in clinical nanomedicine.

Research on the response of bone marrow cells to various nanoparticle types remains limited. A recent report indicated that metallic Ag NPs are toxic to bone marrow cells, causing BM cell cycle arrest and apoptosis. It could be supposed that damage and BM cells will compromise the immune system and increase susceptibility to infection [160]. Although these findings indicate possible hematological risks, the clinical relevance of such effects in humans remains unclear due to insufficient long-term studies evaluating chronic exposure and recovery capacity. In the bloodstream, nanoparticles can induce hemolysis. It has been established that the hemocompatibility of nanomaterials is influenced by a variety of factors, including physicochemical and morphological properties. These properties include, but are not limited to, surface charge, size, geometry, porosity, and specific chemical functionalization. For example, CdTe semiconductor quantum dots (QDs) exert size-dependent toxicity toward red blood cells [161]. This chemotoxicity has been shown to manifest as a variety of symptoms, including but not limited to hemagglutination, the formation of stomatocytes and echinocytes, and hemolysis accompanied by the formation of numerous ghost cells [161]. Induction of hemolysis may cause severe jaundice, heart failure, enlargement of the spleen and liver, and respiratory failure [162]. Other reported complications associated with the administration of nanoparticles include venous thrombosis (Fig. 5). Nemmar et al. demonstrated that intravenous administration of ultrasmall superparamagnetic iron oxide nanoparticles (USPIO) induced thrombosis and cardiac oxidative damage [163]. Such findings may be particularly important for intravenously administered nanocarriers intended for repeated therapeutic use, where vascular safety and thrombogenic potential require careful evaluation.

The liver and kidneys are the primary organs responsible for detoxifying and eliminating xenobiotics, rendering them particularly vulnerable to nanoparticle accumulation and toxicity. Their presence in these organs may lead to inflammation, fibrosis, and impaired function. For instance, silica nanoparticles have been shown to generate ROS, leading to hepatocyte apoptosis [164]. Jakic et al. reported the consequences of the long-term exposure to Au NPs on the structure and function of three vital organs – liver, spleen, and kidneys. The authors reported structural alterations in these organs, including increased macrophage infiltration and elevated fibronectin levels. These findings suggested a toxic response associated with chronic exposure to AuNPs and their slow elimination from tissue [165]. From a translational perspective, these findings raise concerns about the long-term biodistribution and persistence of nanoparticles in human tissues, particularly after repeated administration. However, species-specific differences in metabolism, immune response, and clearance mechanisms significantly complicate direct extrapolation of these findings to clinical settings.

Due to their small size, nanoparticles may not specifically accumulate in the central nervous system. In effect, they can disrupt the blood-brain barrier and induce cerebral ischemia. Neurotoxic effects have been reported for metallic NPs [166, 167], silica NPs [168], and oxide NPs [84, 85]. Although these observations suggest a potential neurological risk associated with NPs exposure, the precise mechanisms governing NPs penetration into the human brain remain incompletely understood. Moreover, current evidence is still insufficient to determine whether such effects occur under clinically relevant conditions.

In conclusion, the toxicity of nanomaterials is closely related to their unique physicochemical properties, such as small size, high surface area-to-volume ratio, reactivity, and morphology. Nanoparticle instability may promote aggregation, alter biodistribution, and increase organ accumulation. Previous studies have shown that nanoparticles may damage the cardiovascular system, induce hepatotoxicity, and contribute to both acute and chronic pathological changes in tissues and organs (Fig. 5). However, most available evidence is derived from in vitro studies, animal models, and review-based analyses conducted under experimental conditions that do not fully reflect human physiology or clinical exposure scenarios. In addition, differences in nanoparticle composition, surface modification, dosage, and routes of administration make direct comparisons between studies difficult and limit the translational value of current data.

Therefore, the potential human implications discussed in this section should be considered indirect predictions rather than direct clinical evidence. Current assumptions regarding human relevance are mainly based on in vitro findings, animal studies, pharmacokinetic analyses, and cross-species toxicological observations, rather than on validated clinical or IVIVE-based models and dedicated procedures. Although these studies provide important insights into potential toxicity mechanisms and safety concerns, further standardized, clinically relevant long-term studies are still required to reliably assess the safety and translational applicability of nanoparticle-based drug delivery systems in humans.

## Effects of nanoparticles on liver enzymatic activity

A great number of drugs, which were tested in clinical trials or marketed to date, have been withdrawn due to hepatotoxicity. Several mechanisms underlie the thus-evoked liver damage: the formation of reactive oxygen species, the production of reactive metabolites, mitochondrial dysfunction, and inflammatory responses. A primary source of these toxic metabolic effects is the Cytochrome P450 (CYP) enzyme family. It may convert relatively non-toxic medicinal substances into reactive metabolites. Therefore, a careful study of drug-metabolizing enzymes is crucial to identify those with particularly adverse and toxic effects on the liver [169].

**Fig. 6:**
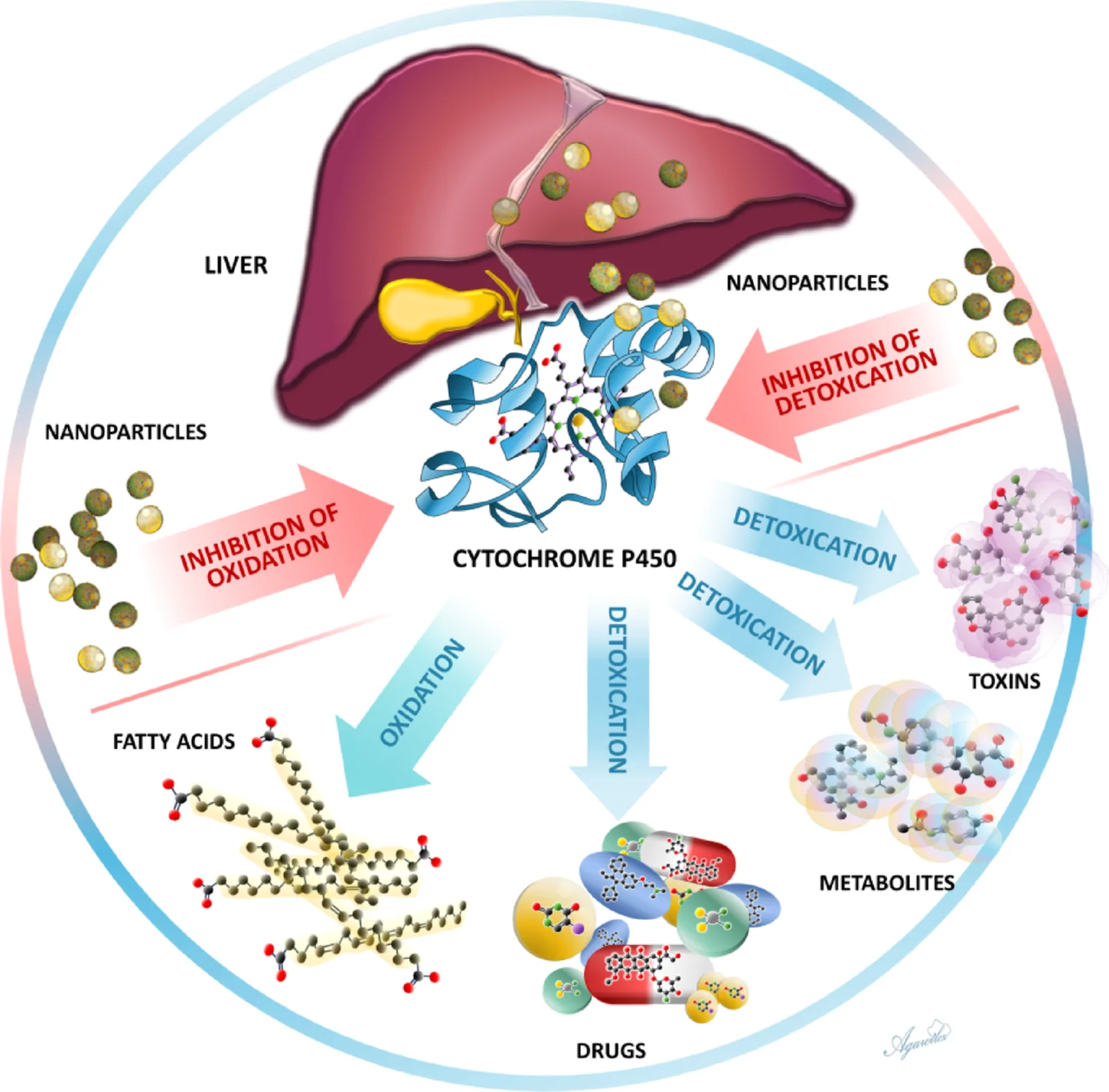
Effect of nanoparticles on the enzymatic activity of cytochrome P450 in the liver. The figure presents the interactions between nanoparticles and hepatic cytochrome P450 enzymes involved in xenobiotic metabolism. Alterations in CYP activity may influence drug biotransformation, metabolic homeostasis, and detoxification, thereby affecting pharmacokinetics and potentially contributing to nanoparticle-associated hepatotoxicity

Cytochromes are proteins involved in electron transport during oxidative phosphorylation. They exist in all human cells, in mitochondria and the endoplasmic reticulum (ER) [170, 171]. They are also abundant in the liver, which plays a central role in metabolism. Cytochromes, which act as monooxygenases, are responsible for oxidizing fatty acids and xenobiotics [172, 173]. They lead to the detoxification of drugs, toxins, and metabolites (Fig. 6). These functions are carried out by three groups of cytochromes: CYP1, CYP2, and CYP3. Interindividual genetic disparities in these enzymes can lead to differences in drug response. CYPs, which metabolize 70–80% of all drugs in phase I metabolism, are expressed in most body tissues [174, 175]. They also function as activators of certain chemotherapeutic agents. Consequently, genetic alterations in CYPs can induce toxicity or lead to inappropriate drug activation. Philips et al. [176] demonstrated that polymorphic forms of P450 enzymes are responsible for adverse drug reactions for 59% of listed drugs [176–178].

In order to evaluate whether given nanoparticles can serve as drug carriers, one has to perform a complete characterization of NPs’ properties (e.g., size, shape, charge, chemical composition). In addition, we shall consider their potential interactions with biological systems. Smaller nanoparticles are favored in drug delivery as they exhibit a higher surface area-to-mass ratio. The latter could enhance their ability to carry therapeutic substances. However, high surface areas also increase their tendency to interact with non-target organs or tissues, potentially disrupting their functions. Ollikainen et al. reported nanoparticles formed by thermally carbonized porous silicon (TCPSi) and its derivatives, aminopropylsilane-modified TCPSi (APTES-TCPSi), alkyne-terminated thermally hydrocarbonized PSi (alkyne-THCPSi). They were shown to act as exogenous inhibitors that adversely affect CYP isoenzyme activity. This interaction can reduce drug efficacy and alter therapeutic outcomes [179]. Key enzymes, CYP3A4 (responsible for the metabolism of approximately 50% of clinically utilized drugs) and CYP2D6 (~ 30%) have been identified as particularly vulnerable. TCPSi-based nanoparticles can inhibit CYP activity by binding directly to the enzyme’s active site. Furthermore, TCPSi NPs can disrupt the mitochondrial membrane lipid bilayer, thereby indirectly affecting the CYPs located within it. This inhibition presents two important issues. First, even slight inhibition of CYPs (Fig. 6) can lead to drug accumulation, resulting in toxic concentrations rather than therapeutic effects. Secondly, if nanoparticle interactions paradoxically accelerate CYP-mediated metabolism, standard drug doses may become ineffective due to overly rapid clearance from the body [179]. Furthermore, CYP enzymes can bioactivate either a drug or its nanocarrier, forming reactive metabolites that modify cellular components and cause damage [180].

Research on CYP modulation has mainly focused on metal nanoparticles, especially gold and silver (AuNPs, AgNPs) [181]. It was demonstrated that AuNPs inhibit CYP3A4, CYP2C19, CYP2C9, and CYP1A2, while AgNPs inhibit CYP3A4, CYP2C19, and CYP2C9. Those enzymes are responsible for over 50% of drug metabolism. Examining the toxicity of AuNPs, Balasubramanian et al. administered a single injection of AuNPs to rats [182]. The results showed a 28-fold increase in CYP1A1 expression and, two months later, a 2-fold increase in CYP3, CYP4, and CYP8 enzymes. Nanoparticle accumulation in the liver also altered lipid metabolism signaling pathways. These findings suggest that targeted therapy with gold nanocarriers may be ineffective due to induced CYP450 activity, resulting in faster metabolism. Additionally, the changes in lipid metabolism can lead to steatosis and hepatitis (Fig. 5).

Ye et al. reported in an in vitro study on liver cells that smaller Au NPs (5–100 nm) had a greater effect on CYP activity, with the impact increasing as the size decreased [183]. Similarly, studies on Japanese rice fish and rainbow trout showed increased CYP1A expression, interpreted as a defense mechanism against the toxic and apoptotic effects likely caused by free radicals from metallic nanoparticles [184, 185]. On the same note, in a study using ~ 180 nm silver nanoparticles orally administered in rats for 2 weeks, no significant effects on CYP were observed. It was concluded that the large particle size and the short exposure time play crucial roles. These results indicate that larger nanoparticles may be safer, even though their drug-delivery efficiency is expected to be lower. Smaller particles are more likely to interact with CYP450 enzymes, leading to cellular toxicity.

Researchers have observed that metallic, hydrophobic silver nanoparticles can compete for the substrate-binding site on CYP, and their size determines whether they can access the catalytic site. Nanoparticles can also inhibit CYP activity by binding to allosteric sites (non-catalytic sites) [186]. CYP enzymes are highly flexible and can change conformation to accommodate ligands in the active site. This flexibility may explain why smaller nanoparticles are more toxic; they can interact with these allosteric sites, including conformational changes that disrupt substrate binding and overall metabolism [187]. The observations indicate that the properties of both the cytochromes and the nanoparticles are fundamental to the experimental outcome, as CYP activity can be altered through direct or indirect mechanisms [188].

## Effects of nanoparticles on gut microbiome

The human microbiome is among the fastest-growing areas of research in biology, medicine, and biotechnology, and its importance extends far beyond the digestive tract [189]. Advances in sequencing and metagenomics technologies have revealed a distinct microbiome ‘fingerprint’ for every single person. Our microbiome is characterized by specific species and functional diversity. It is estimated that the number of genes in microorganisms exceeds that in the human genome by around 150–200 times [190, 191]. The microbiome plays a crucial role in many processes, e.g., metabolism, immune maturation, immune function, gut-brain axis modulation, metabolites production influencing hormone balance and mental health [189]. The imbalance in the microbiome’s composition, dysbiosis, is linked to various chronic conditions. Examples include obesity, type 2 diabetes [192], neurodegenerative diseases (Parkinson’s, Alzheimer’s), autoimmune diseases, mood disorders, atopy, allergies, asthma [193], inflammatory bowel diseases, colon cancer [194] and dermatological conditions (psoriasis, atopic dermatitis). In view of this, the microbiome is considered as a ‘metabolic organ’ of 1–2 kg weight, that plays a pivotal role in maintaining the homeostasis [191].

A large number of reports confirm that nanomaterials (NMs) can penetrate the skin barrier, respiratory epithelium, and gastrointestinal tract [195]. In the context of food and packaging, it is important to note that materials such as metal and non-metal oxides (ZnO, TiO₂, SiO₂) [196], metal nanoparticles (AgNPs) and nanoclays are used directly as additives or as packaging components. This creates a direct route of intestinal exposure [197, 198]. There are also indirect mechanisms that contribute to gastrointestinal exposure. One of them is the ingestion of respiratory tract particles during mucociliary clearance, further linking inhalation and intestinal exposure pathways [199]. There is a growing body of evidence indicating that NMs can alter the composition and function of the intestinal microbiota, affecting both local mucosal homeostasis and overall host physiology [200, 201] (Table 3).

**Table 3:** Nanoparticle-induced alterations of the gut microbiome and associated intestinal and systemic effects in in vivo models

Type of nanoparticles (size)	Animal / route of administration	Impact on the microbiome	Effect on individual bacteria (more/less)	Other intestinal effects and the impact of GM on other systems	Ref
MWCNTs (10.7 ± 3.1 nm)	C57BL/6 mice, oral and inhalation exposure	Disorders of α- and β-diversity, shift in microbiota composition, increase in the *Firmicutes/Bacteroidetes* ratio	↑ *Clostridiales; f*, *↑ Bacteroideaceae*, *↑ Rikenellaceae*, *↑ Helicobacteraceae*, *↑ Anaeroplasmataceae*, *↓ Prevotellaceae*, *↓ Proteobacteria*, *↑ Tenericutes*	Dysbiosis modulates the immune response of the lungs, reduction in the severity of inflammation with depletion of the microbiota	[201]
SWCNTs (diameter 1.04–1.17 nm)	ICR mice, oral exposure for 7 days, dose 2.5 mg/kg/d	Microbiome disturbances; dose- and time-dependent changes; increased intestinal permeability	*↑ Bacteroidetes*, *↑ Proteobacteria;* *↓ Firmicutes*, *↓ Actinobacteria*	↑ IL-1β, IL-6, TNF-α; increased histological score; CNT deposition in the colon lumen; microvillus and crypt damage; hematological and biochemical changes	[202]
GO (236 ± 5 nm in water, 766 ± 179 nm in simulated intestinal fluid)	Mature ICR mice, dose 40 mg/kg/d p.o. on GD7–16	Reduced α- and β-diversity of the microbiome, increased *Firmicutes/Bacteroidetes* ratio	*↑ Euryarchaeota*, *↑ Methanobrevibacter*, *↑ Sellimonas;* *↓ Cyanobacteria*, *↓ Chloroflexi*, *↓ Latescibacteria*, *↓ Fusobacterium*, *↓ Phascolarctobacterium*, *↓ Aneurinibacillus*, *↓ Gammaproteobacteria*, *↓ Corynebacterium*	Morphological changes in the intestine: irregular arrangement of epithelial cells, increased intestinal permeability, increased inflammation (↑ IL6), decreased Claudin1 and Muc2 mRNA	[203]
GR (0.508 μm × 1.58 nm)	Zebrafish (> 6 months, AB strain), diet containing GR for 21 days	Change in microbiome structure, decrease in diversity, oxidative stress (↓ SOD, ↑ GPx)	*↑ Fusobacteria*, *↑ Cetobacterium*, *↑ Lactobacillus;* *↓ Firmicutes*, *↓ Pseudomonas*, * ↓unidentified Erysipelotrichaceae*	Increased vacuolization, ↑ number of goblet cells, changes in intestinal morphology, change in antioxidant enzyme activity	[204]
GO (0.297 μm × 1.76 nm)	Zebrafish (> 6 months, AB strain), diet containing GO for 21 days	Changes in the structure of the microbiome, oxidative stress (↓CAT, ↑GPx)	*↑ Fusobacteria*, *↑ Cetobacterium*, *↑ Lactobacillus;* *↓ Firmicutes*, *↓ Pseudomonas*, *↓ unidentified Erysipelotrichaceae*	Increased vacuolization, ↑ number of goblet cells, changes in intestinal morphology, change in antioxidant enzyme activity	[204]
rGO (41.5 nm × 13 nm)	Zebrafish (> 6 months, AB strain), diet containing rGO for 21 days	Change in microbiome structure, oxidative stress (↑GPx)	*↑ Fusobacteria*, *↑ Cetobacterium*, *↑ Lactobacillus (less than GR/GO);* *↓ Firmicutes*, *↓ Pseudomonas*, *↓ unidentified Erysipelotrichaceae;* *↓ Aeromonas;* *↑ Cetobacterium 3*,*17×*	Disruption of intestinal cell borders, increased vacuolization, ↑ number of goblet cells, and a change in antioxidant enzyme activity	[204]
GR (thickness 0.5–3 nm; flakes 400–800 nm)	ICR mice, oral exposure for 28 days, doses of 1, 10, 100 mg/kg/d	Increased diversity of the microbiome; shift in structure toward Gram-negative dominance	*↑ Prevotella*, *↑ Paraprevotella*, *↑ Anaeroplasma;* *↓ Lactobacillus*	Increase in the number and diversity of antibiotic resistance genes (ARGs); elevated genes for oxidative stress response and cell membrane integrity; toxicity mechanism related to damage to bacterial membranes	[9]
CNF (~ 50 nm)	Wistar Han rats, oral exposure for 5 weeks (10 administrations via feeding tube), 1% in water or cream	No acute toxicity; modulation of the gut microbiome, no significant changes in the fecal metabolome	*↓ SCFA-producing species: Coprococcus catus*, *Bacteroides acidifaciens*	↑ insulin and serum IgA production; changes in the expression of cell junction genes in the ileal epithelium; an increase in cytokines modulating CD8 T cell proliferation	[14]
SNP ( 48.53 nm)	Mice, oral exposure	Marked microbiome dysbiosis	*↑ Bacteroidetes*, *↑ Alloprevotella*, *↑ Bacteroides;* *↓ Firmicutes*	Microbiome disorders related to the immune system, circulation, and lungs	[205]
Ag NPs (60–150 nm)	C57BL/6J mice, oral exposure, (0.5 and 2.5 mg/kg), 14–28 days	After 14 days: decrease in microbiome diversity, change in microbiota structure. After 28 days: partial restoration of the microbiome (alpha diversity and PCA close to control)	*↓ Bacteroidales-S24-7 (Bacteroidetes)*, *↑ Lachnospiraceae (Firmicutes);* Gram-negative more sensitive	Intestinal metabolome disorders (↑ 1 H-indole-3-carboxylic acid, ↑ 5-HT in the intestines and blood), modulation of metabolic pathways associated with the nervous system; protein “crown” reduces the toxicity of Ag⁺ ions	[206]
Ag NWs (1–2 μm)	C57BL/6J mice, oral exposure, (0.5 and 2.5 mg/kg), 14–28 days	Similar pattern of changes as Ag NPs, but weaker effect; partial restoration of the microbiome after 28 days	*↓ Bacteroidales-S24-7 (Bacteroidetes)*, *↑ Lachnospiraceae (Firmicutes);* smaller changes than in Ag NPs	Intestinal metabolome disturbances, partially permanent; microbiome self-regulation mechanisms limit toxicity	[206]
Ag NPs (15 ± 5 nm)	C57BL/6 mice, oral exposure, long-term 120 days, various doses	Initial decrease in alpha diversity (21 days), gradual recovery and surpassing of controls after 120 days; systematic remodeling of the microbiome; increase in the *Firmicutes/Bacteroidetes* ratio	*↓ Bacteroidetes*, *↑ Firmicutes; disturbed genera: ↑ Turicibacter*,* Ruminococcus_1*,* ↑Rikenellaceae_RC9_gut_group↑Eubacterium_coprostanoligenes_group*,* Anaeroplasma*	Disorders of microbiome metabolic functions: energy, carbohydrate, and lipid metabolism; stimulation of metabolic pathways (energy, purines, amino acids, lipids, genetic processes); correlation with changes in liver metabolites; dysbiosis mediates the metabolic effects of nanotoxicity	[207]
Ag NPs (7 nm)	Wistar rats, males, oral exposure, 28 days (100 mg/kg)	Significant changes in the gut microbiome, a decrease in the *Firmicutes/Bacteroidetes* ratio	*↑ Bacteroidota*, *↓ Akkermansia (Verrucomicrobia)*, *↓ Proteobacteria (Morganellaceae*,* Proteus)*, *↑ Ruminococcaceae*, *↑Oscillospiraceae*, *↑ Prevotellaceae*, *↓ Lactobacillus*, *↑ Erysipelotrichales*	Reduction in protective species, potentially pro-inflammatory effect of the microbiome	[208]
Ag NPs (~ 50 nm)	zebrafish, males and females, exposure 35 days, concentrations 10, 33, 100 µg/L	Gender-dependent intestinal dysbiosis; in males, a decrease in microbiome richness and diversity; in females, no changes	Males: *↑ Fusobacteria*, *↑ Cetobacterium (7→37–57%)*, *↑ Aeromonas (at the highest concentration);* *↓ Proteobacteria (inversely correlated with Fusobacteria).* Females: *no significant changes*	The structure of the male microbiome significantly altered; females resistant to changes; the effect clearly sex-dependent	[209]
Ag NCs (cubic, 50 nm)	Male Sprague–Dawley rats, oral exposure for 14 days	Microbiome dysbiosis; no changes in α-diversity	*↓ Clostridium spp.*, *↓ Bacteroides uniformis*, *↓ Christensenellaceae*, *↓ Coprococcus eutactus*	Impaired fiber metabolism, SCFA production, and intestinal barrier integrity; impaired metabolic pathways (lipids, carbohydrates, amino acids, energy)	[209]
Ag NSs (spherical, 45 nm)	Male Sprague–Dawley rats, oral exposure for 14 days	Microbiome dysbiosis; no changes in α-diversity	*↓ Oscillospira spp.*, *↓ Dehalobacterium spp.*, *↓ Peptococcaceae*, *↓ Corynebacterium spp.*, *↓ Aggregatibacter pneumotropica;* *↑ Anaerostipes spp.*, *↑ Rikenellaceae*, *↑ Dehalobacteriaceae*	Disruption of protein and monocarbon metabolism; changes in metabolic pathways (lipids, carbohydrates, amino acids, energy)	[210]
Ag NPs (12.2 ± 3.0 nm)	ICR male mice, oral exposure for 7 days, dose 2.5 mg/kg/d	Intestinal dysbiosis; decrease in *Firmicutes/Bacteroidetes* ratio	*↓ Lactobacillus;* increase in the proportion of rare bacterial families	Symptoms of colitis: ↑ disease activity index, histological changes, damage to microvilli and tight junctions, ↑ pro-inflammatory cytokines (IL-1β, IL-6, TNF-α)	[211]
Ag NPs (55.17 ± 2.67 nm)	Male C57BL/6 mice, oral exposure for 28 days	Dose-dependent dysbiosis; impaired α- and β-diversity; ↑ *Firmicutes/Bacteroidetes* ratio	*↑ Lachnospiraceae;* *↓ Bacteroidetes (family S24-7)*	No general toxicity or obvious tissue damage; effect dependent on feed freshness and Ag NPs sulfidation	[8]
TiO₂ NPs (30 ± 7 nm)	Mice, long-term oral exposure; standard and high-fat diet	Severe dysbiosis; increase in α-diversity; strong increase in the *Firmicutes/Bacteroidetes* ratio (> 3×)	*↑ Firmicutes;* *↑ Desulfovibrionaceae*, *↑ Oscillospira*, *↑ Coprococcus*, *↑ Ruminococcus*, *↑ Ruminococcaceae*, *↑ Lachnospiraceae;* *↓* some Gram-negative bacteria	Damage to the mucus layer; ↑ intestinal permeability; ↑ LPS in the intestines and blood; low-grade inflammation; microbiota necessary for pro-obesity and pro-inflammatory effects	[10]
TiO₂ NPs (29 ± 9 nm)	Male Sprague–Dawley rats; oral exposure for 90 days	Dose-dependent dysbiosis; increase in microbiota richness and diversity (number of species and phylogenetic diversity); decrease in the *Firmicutes/Bacteroidetes* ratio	*↑ Lactobacillus reuteri;* *↓ Romboutsia*	↑ LPS; pro-inflammatory stress; disruption of microbiota metabolic functions; nanotoxicity features	[212]
TiO₂ NPs (30–300 nm)	Male C57BL/6J mice; oral exposure for 21 days; doses of 10–50 mg/kg bw/day	No changes in α-diversity; dysbiosis at the taxon level and interspecies interactions	*↑ Parabacteroides*, *↑ Lactobacillus*, *↑ Allobaculum;* *↓ Adlercreutzia*, *↓ unclassified Clostridiaceae*	Changes in colonic epithelial gene expression (↓ *Muc2*, ↑ beta-defensin); shortening of crypt length; ↑ macrophages and CD8⁺ T lymphocytes; ↑ proinflammatory cytokines (IL-6, TNF-α, IL-17 A); disruption of intestinal homeostasis	[213]
TiO₂ NPs (10, 50, 100 nm)	Female C57BL/6J mice; oral exposure for 2–3 months	No changes in α-diversity; taxonomic dysbiosis (↑*Bacteroidetes*, ↓*Actinobacteria*)	*↓ Bifidobacterium; * *↓Lactobacillus (including L. johnsonii)*	Symptom severity in a mouse model of colitis (DSS-colitis): poorer recovery after DSS, colon shortening, enlarged spleen; immune disorders (↓ CD4+, ↓ Treg, ↓ macrophages)	[12]
TiO₂ NPs (25, 50, 80 nm)	Adult male C57BL/6 mice; oral exposure for 7 days	Dysbiosis associated with degradation of the mucosal barrier; microbiota as a key mediator of toxicity (confirmed by FMT)	*↓ Bifidobacterium (*in colon contents and feces*)*	Damage to the colonic epithelium; ↓ mucus layer thickness; ↓ tight junction proteins (ZO-1, occludin); FMT transfers phenotype; inulin protects the intestinal barrier	[214]
TiO₂ NPs (~ 40 nm)	In vitro: probiotics exposed to 125–500 µg/ml. In vivo: white albino mice, 50–100 µg/day/mouse orally	Potent inhibition of probiotic growth and survival; dysbiosis consisting of a reduction in beneficial bacteria	*↓ Bacillus coagulans* *↓ Enterococcus faecalis* *↓ Enterococcus faecium*	↓ Biofilm formation; ↓ metabolic activity (MTT); ↓ viability (Live/Dead); ↓ bacterial count in qPCR; predicted microflora disturbances in children after consuming 20 g of chocolate	[13]
TiO₂ NPs (29 ± 9 nm)	Sprague-Dawley rats, oral exposure for 30 days; doses 0, 2, 10, 50 mg/kg/d	Intestinal dysbiosis; changes in microbiome structure	*Lactobacillus gasseri*,* Turicibacter*,* L. NK4A136_group;* *↓ Veillonella*	Morphological changes in the colon: inflammatory infiltrates, mitochondrial abnormalities; changes in 25 metabolites (including ↑ N-acetylhistamine, caprolactam, glycerophosphocholine; ↓ 4-methyl-5-thiazoloethanol, L-histidine, L-ornithine); dysregulation of aminoacyl-tRNA biosynthesis; ↑ LPS → oxidative stress and inflammatory response	[215]
TiO₂ NPs (20–420 nm; 16–361 nm, depending on the environment)	Male C57BL/6 mice; oral exposure for 28 days	Crystal phase-dependent dysbiosis; rutile caused stronger modulation of the microbiome	Rutile: *↑ Proteobacteria*, *↑ Firmicutes*, *↑ Actinobacteria;* *↓ Bacteroidetes;* *↑ Rhodococcus*, *↑ Escherichia-Shigella;* *↓ Barnesiella*, *↓ Prevotella*, *↓ Turicibacter*, *↓ Lactobacillus.* Anatase: *↑ Verrucomicrobia (Akkermansia)*, *↑ Bacteroides;* *↓ Barnesiella*, *↓ Prevotella*, *↓ Turicibacter*, *↓ Lactobacillus*	Metabolic dysfunction of the microbiota; chronic oral exposure reveals features of nanotoxicity; effect dependent on the crystal phase.	[216]
ZnO NPs (< 50 nm)	C57BL/6J male mice, oral exposure for 30 days, 26 mg/kg	Structural remodeling of the microbiome, α-diversity unchanged	*↑ Actinobacteria;* *↑ Bifidobacterium*, *↑ Adlercreutzia*, *↑ Sutterella;* *↑ Bifidobacteriaceae*	Neurobehavioral disorders: impaired learning and memory, inhibition of locomotor activity; serum and hippocampal metabolism disorders; changes in neuronal gene expression (*Bdnf*,* Dlg4*); microbiota–metabolites–neuronal gene correlations	[217]
ZnO NPs (no exact size specified)	Weaned piglets, oral exposure for 14 days (600 mg Zn/kg feed)	Segment-specific disturbances in the composition and diversity of the microbiome; destabilization of the bacterial ecosystem	Ileum: *↓ Lactobacillus*, *↑ Streptococcus*, *↑ Bacillus*, *↑ Halomonadaceae.* Cecum: *↓ OTU*, * ↓ Chao1*, * increase in unidentified bacteria* *.* Colon: *↑ Firmicutes*, *↓ Bacteroidetes;* *↓ Prevotella*, *↓ Oscillospira*, *↑Lactobacillus*, *↑Streptococcus*	Disruption of microbiome metabolic functions (decrease in SCFA producers: *Ruminococcus*,* Dorea*,* Roseburia*); disruption of the microbiota-immunity axis (↓ IFN-γ, IL-1β, TNF-α, NF-κB); risk of inflammation and intestinal barrier disruption	[218]
SiO₂ NPs (~ 14.3 nm)	Wistar rats, males, oral exposure, 28 days (100 mg/kg)	Significant changes in the gut microbiome, an increase in the *Firmicutes/Bacteroidetes* ratio	*↑ Firmicutes*, *↑ Akkermansia (Verrucomicrobia)*, *↓ Proteobacteria (Morganellaceae*,* Proteus)*, *↑ Oscillospiraceae*, *↑ Ruminococcaceae*, *↑Lachnospirales/Lachnospiraceae* *↓ Turicibacter*, *↓ Erysipelotrichaceae*, *↓ Peptostreptococcales-Tissierellales*	Reduction of certain protective species, changes that may be associated with inflammation	[208]
SiO₂ NPs (10.8 ± 1.7 nm)	ICR male mice, oral exposure for 7 days, dose 2.5 mg/kg/d	Increased microbiota richness and diversity	*↑ Lactobacillus*	↑ Proinflammatory cytokine levels (IL-1β, IL-6, TNF-α) in the colon	[211]

Ag NCs – Silver nanocubes, Ag NPs – Silver nanoparticles, Ag NWs – Silver nanowires, Ag NSs – Silver nanospheres, CNF – Cellulose nanofibers, GO – Graphene oxide, GR – Graphene; MWCNTs – Multi-walled carbon nanotubes, IL- Interleukin, LPS – Lipopolysaccharide, rGO – Reduced graphene oxide, CFA – Short-chain fatty acids, SiO₂ NPs – Silica dioxide nanoparticles, SNP – Silica nanoparticles, SWCNTs – Single-walled carbon nanotubes, TiO₂ NPs – Titanium dioxide nanoparticles, ZnO NPs – Zinc oxide nanoparticles

Summary of reported effects of nanoparticle exposure on gut microbiota composition and gastrointestinal homeostasis observed in experimental studies. The table presents representative bacterial taxa and biological processes affected by nanoparticle-based systems, including microbiome dysbiosis, altered intestinal permeability, inflammatory responses, oxidative stress, and changes in bacterial abundance. ↑ increased / ↓ decreased abundance, activity, expression/supression, permeability, or biological response. The observed microbiome-related effects may vary depending on nanoparticle composition, size, surface functionalization, administered dose, exposure duration, and experimental model

**Fig. 7:**
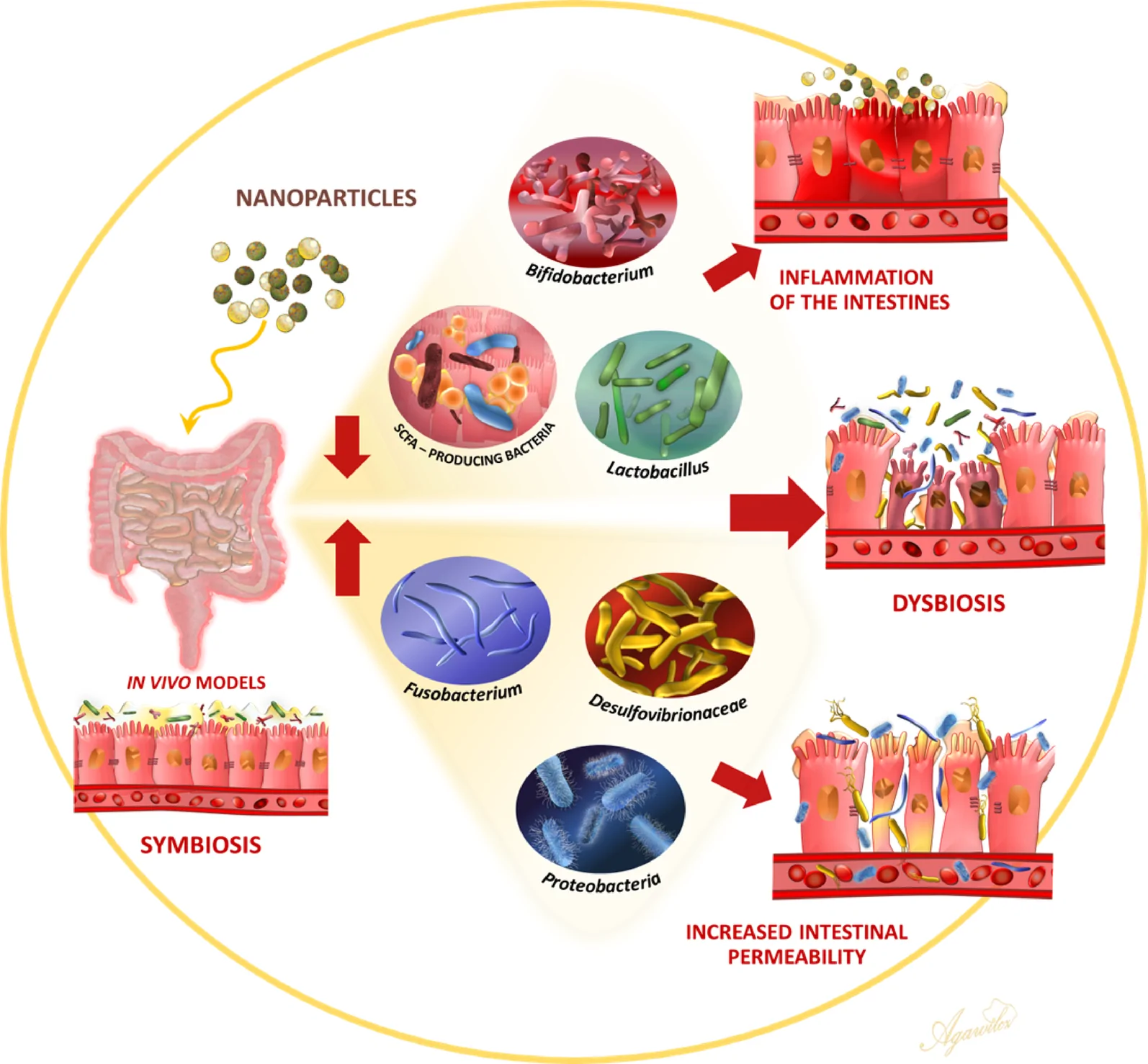
Schematic overview of nanoparticle-induced alterations in the gut microbiome and their consequences for intestinal homeostasis. Nanoparticle exposure may disrupt intestinal microbial homeostasis and alter the composition and metabolic activity of the gut microbiota. These disturbances are associated with impaired intestinal barrier integrity, altered short-chain fatty acid production, inflammatory activation, and dysregulated gut–immune interactions, potentially contributing to systemic metabolic and immunological alterations

Animal models are used to examine the influence of nanoparticles on the gut microbiome. It was shown that oral exposure can lead to intestinal dysbiosis (Fig. 7). The final outcome depends on the nanomaterial type, size, dose, and the animal model used. Researchers have examined many different nanomaterials in this context. Silver has been studied in various forms, including AgNPs, AgNWs, AgNCs, and AgNSs [207, 208, 210–212, 220]. Titanium dioxide (TiO₂ NPs) has also been investigated [10, 12, 214–216]. Other examples include zinc oxide (ZnO NPs) [218, 219] and silica (SiO₂ NPs) [209, 212]. Carbon nanotubes, both single-walled (SWCNTs) and multi-walled (MWCNTs), have also been examined [202, 203]. Graphene (GR) and its derivatives, graphene oxide (GO), reduced graphene oxide (rGO), and graphene quantum dots (GQDs) [9, 204, 205], are also on the list. Even nanocellulose (CNF) [14] has been studied. All of them alter the composition and diversity of the gut microbiota. The beneficial probiotic bacteria, such as Lactobacillus, Bifidobacterium, and Faecalibacterium, typically decline. At the same time, pro-inflammatory species, *Proteobacteria*, *Enterobacteriaceae*, and *Desulfovibrio* [11] advance (Fig. 7). These changes weaken the intestinal barrier, disrupt SCFA metabolism, and enhance inflammatory responses. Particle size determines how far the damage spreads. Smaller particles (10–30 nm) penetrate deeper into the gastrointestinal tract and thus affect the microbiome. Larger ones (50–100 nm), in turn, have more localized effects or affect specific bacterial strains.

In the case of Ag NPs, rapid declines in microbiome diversity were observed, followed by partial recovery after several weeks. Smaller particles (7–60 nm) exerted stronger dysbiotic effects, altering intestinal metabolism and elevating levels of 5-HT and 1 H-indole-3-carboxylic acid. The latter has been associated with modulation of neural pathways [8, 207, 208, 210, 211]. Depending on their type and size (GO, rGO, GR and GQDs), graphene nanoparticles caused a decrease in α- and β-diversity, reflecting reduced richness and evenness of microorganisms in individual intestinal samples (α-diversity) and altered community composition between the samples studied (β-diversity), as well as an increase in the abundance of bacteria that produce short-chain fatty acids, which may have positive metabolic effects [9, 204, 205].

In mouse models, carbon nanotubes (SWCNTs and MWCNTs) altered the *Firmicutes/Bacteroidetes* ratio and the composition of bacterial families and genera. These changes affected the lung immune response, indicating the existence of a gut-lung axis [202, 203]. It was demonstrated that titanium- and silicon-based nanoparticles can induce dysbiosis, compromise mucosal barrier integrity, and increase intestinal permeability. When these NPs, such as TiO₂ and SiO₂, are combined with a high-fat diet and/or other environmental factors, they may exacerbate pro-inflammatory responses and contribute to metabolic disturbances linked to obesity [10, 12, 209, 212, 214–216]. In contrast, ZnO NPs, especially at lower doses, have been shown to increase the abundance of beneficial probiotic bacteria and to support protective functions of the intestine. However, if exposure occurs at higher doses, it may disrupt SCFA metabolism and perturb the microbiota-immune axis [218, 219].

All nanomaterials that were analyzed showed effects that were dependent on the sex of the animal on the exposure times [210, 221]. These dysbiotic changes were associated with disturbances in metabolism, immunity, and neurobehavior. This highlights that the gut microbiome is a key mediator for nanotoxicity. Changes in the composition of the gut microbiota could serve as early indicators of nanomaterial toxicity. Therefore, they should be integrated into safety assessments for consumer and pharmaceutical products. The effects that are most frequently observed include reductions in probiotic bacteria which are beneficial, and increase in taxa that are potentially pro-inflammatory, which can result in dysbiosis, disturbances in SCFA metabolism, and increased intestinal permeability (Fig. 7). Collectively, these observations underscore that the gut microbiome has value as a sensitive indicator for nanotoxicity, and they support its inclusion in both safety evaluations and also in further studies on interactions between nanomaterials and the host.

## The toxic effect of nanoparticles on the reproductive system

The global prevalence of infertility is rising, with approximately 17.5% of adults experiencing this condition at some point in their lives. This is according to what the World Health Organization says [222]. Infertility, which is a condition affecting either the male or female reproductive system, is defined as the failure to achieve pregnancy after 12 months of regular unprotected intercourse. For women, the most important causes include tubal obstruction. This happens because of sexually transmitted infections that were not treated. Also, complications that arise from abortion or from childbirth are important [223]. Additionally, there are ovarian disorders such as polycystic ovary syndrome (PCOS). This condition is the most common cause of anovulation [224]. Furthermore, inflammatory disorders of the uterus, for example, endometriosis, congenital defects like a uterine septum, or fibroids, which can make it difficult for an embryo to implant, and also hormonal disorders that affect the balance of hormones, are all important causes of infertility [223].

In men, the main factors are obstruction of the reproductive tract, causing ejaculation disorders. Hormonal disorders in the secretion of testosterone regulate sperm production, testicular failure in sperm production, for example, due to varicocele, and abnormal sperm function and quality [223].

With the development of civilization, the incidence of infertility is increasing. Lifestyle factors like smoking, heavy drinking, and obesity are key here. In addition, exposure to environmental pollutants and toxins can directly harm gametes (eggs and sperm), reducing their numbers and degrading their quality. Fertility can also be affected by trauma, reproductive tract infections, pituitary or gonadal tumors, as well as drug therapies [223].

Since the integrity of reproductive processes, including fertility, reproductive capacity, and embryonic development, is essential for the survival of species, it is crucial to raise scientific awareness of the toxic effects of nanoparticle exposure on reproductive health [23]. As reported by Souza et al., in nanotoxicological studies conducted to date on mammals as experimental models, inorganic nanoparticles dominated (88%), while carbon nanomaterials accounted for 10% and organic nanoparticles only 2% [225]. The intensive use of nanomaterials in products for consumers [226], when combined with their significant production at the global level [227], results widespread exposure for humans. This necessitates a detailed study of their potential impact on reproductive functions [228]. The body’s first line of defence against toxins is its biological barriers. In the reproductive system, these include the blood-testis barrier, the placental barrier, and the epithelial barrier. However, because of their small size, nanoparticles can penetrate these barriers and accumulate in the gonads. This includes testes, epididymis, ovaries, and uterus. Studies indicate that Ag NPs can have toxic effects on reproductive system cells and also on cells that support their development. These include primary and secondary follicles, germ line stem cells, Sertoli cells, and Leydig cells [16, 17]. Furthermore, as indicated by Xu et al., Ag NPs can modulate reproductive behavior by affecting hormone secretion within reproductive organs and glands. The authors emphasize that the toxic effects of Ag NPs on the reproductive system are multifactorial, including enhanced inflammation, DNA damage, downregulation of genes essential for reproductive cell function, mitochondrial dysfunction, increased ROS production, and the induction of apoptosis [18].

### Effects on the male reproductive system

**Fig. 8:**
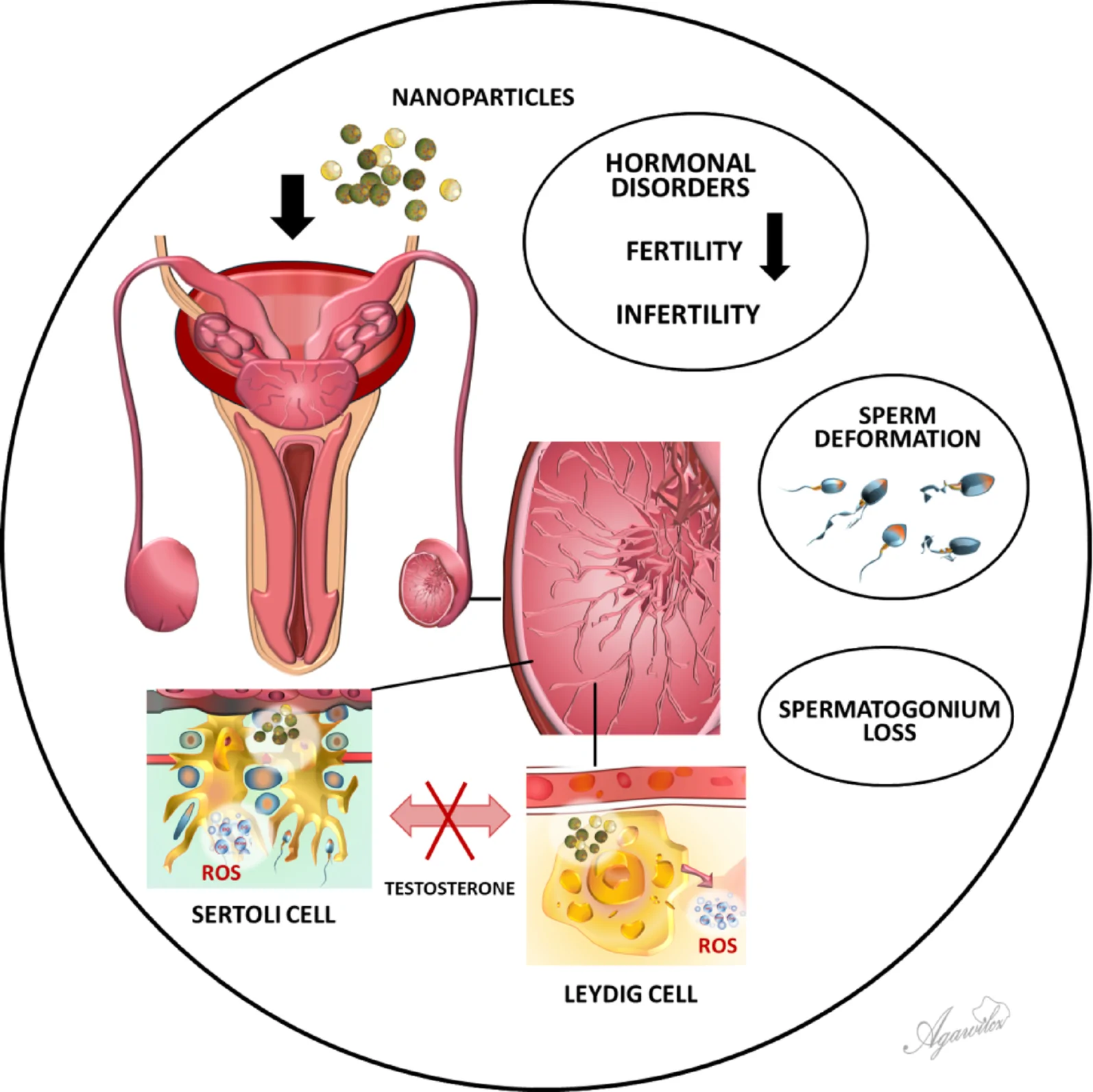
Schematic representation of nanoparticle-induced toxicity in the male reproductive system. The figure illustrates the potential impact of nanoparticle exposure on male reproductive function. Alterations in spermatogenesis, testosterone synthesis, and the physiological activity of Sertoli and Leydig cells may contribute to reduced sperm quality, impaired reproductive capacity, and disturbances in male fertility following nanoparticle accumulation within reproductive tissues

Previous studies clearly indicate that exposure to nanoparticles can disrupt the function of the male reproductive system. This occurs in a dose-dependent manner and depends on the physicochemical properties of the nanoparticles and the type of experimental model used (Fig. 8). The size of nanoparticles influences the toxicity of Ag NPs, with reproductive cells showing variable sensitivity. In particular, particles of 10 nm demonstrate cytotoxicity that is significantly greater than particles of 20 nm. This emphasizes the role of particle size and of cell type in determining what the biological effects will be. Exposure to these nanoparticles reduces the viability and proliferation of Leydig and Sertoli cells. It also leads to damage to the membrane and to the mitochondrial dysfunction (Fig. 8). All of this is accompanied by the accumulation of nanoparticles in the cytoplasm and cell nucleus. These phenomena were accompanied by increased ROS production, activation of the p53, p38, and pErk1/2 signaling pathways, and decreased expression of genes involved in testosterone synthesis and tight-junction integrity. Inhibition of spermatogonial stem cells’ proliferation and self-renewal was also observed, with significant implications for long-term fertility maintenance [16] (Fig. 8). Furthermore, exposure to Ag NPs disrupts the maintenance of germline stem cells in the Drosophila testis, potentially affecting fertility [17].

Similar results were observed for other inorganic nanoparticles. ZnO NP, TiO₂ NPs, and SiO₂ NPs induced oxidative stress, DNA damage, and apoptosis in Sertoli and Leydig cells. These effects coexisted with decreased activity of antioxidant enzymes (SOD, CAT, GPx), increased lipid peroxidation, and decreased Sertoli cell viability. In the case of ZnO NPs, a decrease in the CYP17A1 and CYP1B1 expression was also observed, indicating disturbances in the hypothalamic-pituitary-testicular axis and reduced testosterone synthesis in both in vitro and in vivo models. This is supported by experimental studies showing that ZnO NPs increase testicular inflammation and reduce the viability of reproductive cells, potentially affecting male fertility [19]. Review analyses also indicate that various metallic oxide nanoparticles (e.g., ZnO, TiO₂, Al₂O₃, Fe₂O₃) can disrupt male reproductive system function through oxidative stress and changes in testicular structure [229]. These mechanisms have been confirmed in various organism models. In *Drosophila melanogaster*, Ag NPs accumulated in the gonads at concentrations of 0–5 µg/mL over 7 days. This resulted in reduced survival, delayed development, and decreased male fertility. At the same time, there was a decrease in the number of germline stem cells (GSCs) and their premature differentiation [17]. Literature reviews indicate that nanoparticles can persist in testicular tissue for prolonged periods, increasing the risk of long-term toxic effects [230]. ZnO and TiO₂ NPs induce spermatogonial loss and impair spermatogenesis, resulting in decreased sperm count and motility. At the cellular level, these nanoparticles cause mitochondrial dysfunction and trigger apoptosis. Increased ROS production suggests that oxidative stress plays a key role in mediating these effects [22].

Some rodent studies provide evidence of a significant impact of NPs on fertility. Subchronic oral exposure to Ag NPs stabilized with poly(vinylpyrrolidone) (PVP-AgNPs) (50–200 mg/kg/day for 14–90 days) resulted in dose-dependent abnormalities in sperm morphology. These included deformities of the head, kinks in the tail, and loss of the head itself (Fig. 8). These changes were accompanied by decreased motility and viability of sperm [231]. Other studies have shown that ZnO, TiO₂, SiO₂, Al₂O₃, and Fe₂O₃ nanoparticles can cross the blood-testis barrier, accumulate in the nucleus, and lead to degeneration of the seminiferous tubules. They also cause necrosis of Sertoli cells and disorders of the spermatogenic epithelium. All of this was accompanied by increased levels of MDA and by decreased activity of the antioxidant enzyme. This confirms that oxidative stress is a key mechanism underlying the toxic effects of nanoparticles on the male reproductive system [22]. Furthermore, studies have demonstrated that ZnO NPs exhibit a dose-dependent effect: increased exposure is associated with decreased testosterone concentration and sperm count. This indicates that reproductive function is impaired [232].

Previous studies clearly indicate that exposure to metallic and oxide nanoparticles can significantly disrupt the functioning of the male reproductive system. The specific effects that nanoparticles have depend on various factors. The latter includes the type of particles and their size, the administered dose, and the experimental model used. Such effects include functional impairments. Examples of this are reduced viability and reduced proliferation of Leydig and Sertoli cells. They also include alterations that are mechanistic, such as dysfunction of the mitochondria, oxidative stress, damage to DNA, apoptosis, and changes in how genes that are involved in testosterone synthesis and tight junction integrity are expressed (Fig. 8). Mechanisms that are similar have been observed in models that are both in vitro and in vivo (including mammals and *Drosophila*). The effects include reduced sperm count and motility, as well as the potential for long-term impairment of male fertility. These findings highlight the importance of further research on nanoparticle toxicity and the need for caution in their biological and medical applications.

### Effects on the female reproductive system

**Fig. 9:**
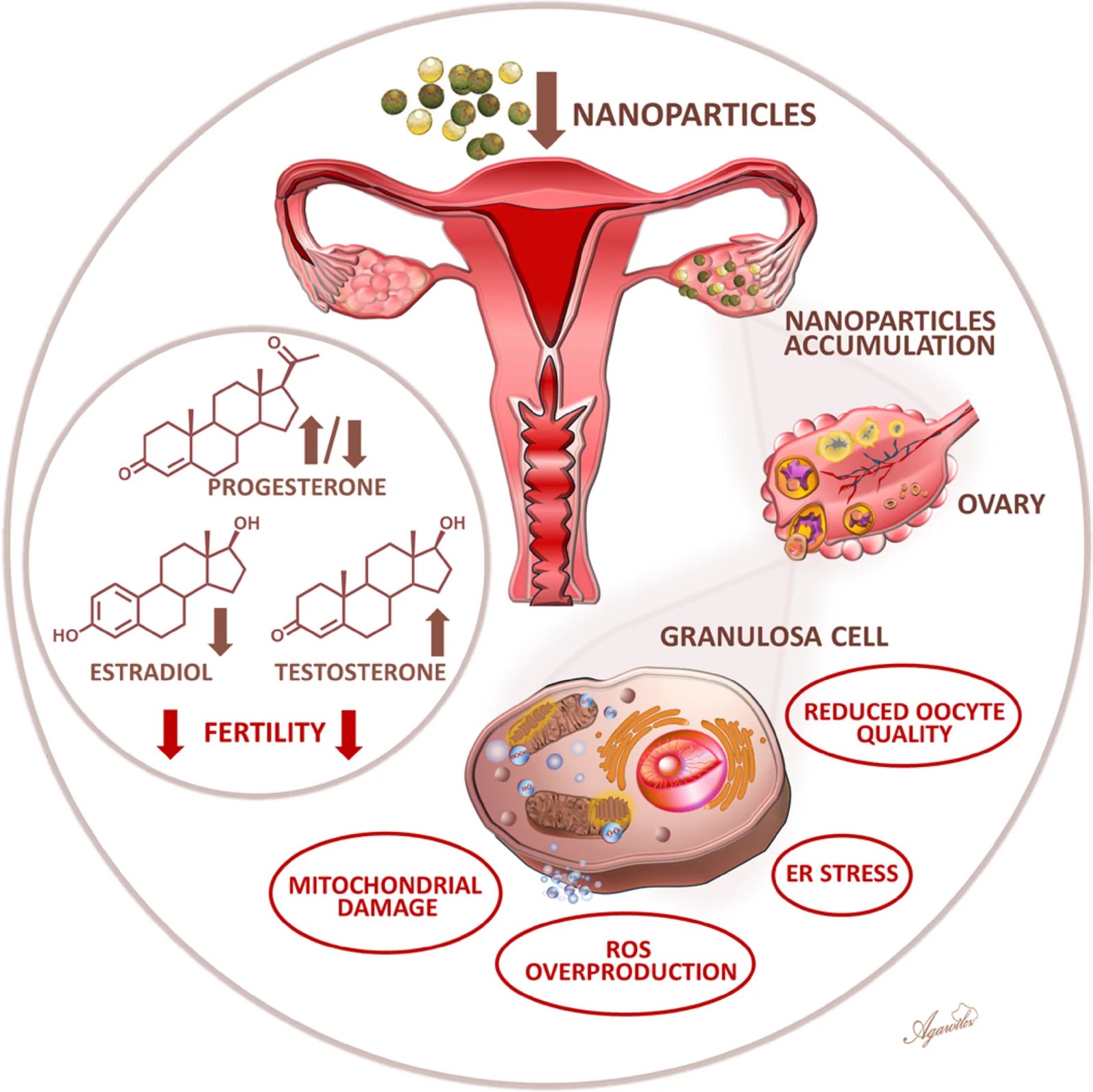
Schematic representation of the effects of nanoparticles on the female reproductive system. Nanoparticle accumulation within female reproductive tissues may interfere with ovarian function, follicular development, and hormonal balance, potentially contributing to impaired fertility and adverse reproductive outcomes. The presented mechanisms highlight the possible influence of nanoparticle exposure on reproductive homeostasis and female reproductive health

The pool of ovarian follicles in women is limited, and only a fraction of these follicles are able to reach maturity and to ovulate throughout the reproductive years. This results in a relatively constrained reproductive capacity [233]. The female reproductive system, which includes the ovaries and the uterus, undergoes cyclical processes of growth, maturation, and regeneration. These processes are tightly controlled by hormones. Endocrine homeostasis in the female reproductive system is sensitive to pathophysiological influences. Exogenous nanoparticles may disrupt normal functioning. This can lead to impairments in reproduction and in the development of the fetus [20] (Fig. 9).

When mice were orally exposed to TiO₂ nanoparticles at doses of 1.25 to 5 mg/kg for 60 days, this led to a marked reduction in the number of primary follicles, secondary follicles, and antral follicles. The corpus luteum was also reduced, and this was accompanied by an increase in follicles that are atretic. Mechanistically, these effects were linked to the upregulation of growth- and apoptosis-related factors. These factors include TGF-β1, TGF-βR1, PTEN, and Foxo3a. There was also downregulation of proteins that are important in the PI3K/AKT/mTOR signaling pathway and in the AKT/p70S6K rpS6/TSC/mTOR signaling pathway. When all of this is considered together, these findings indicate that TiO₂ NPs have the ability to inhibit follicular development. They do this by disrupting TGF-β signaling and pathways important for cell growth and survival [234].

Other mechanisms of nanoparticle toxicity have also been observed in mammalian models. Studies conducted in ICR mice have shown that TiO₂ NPs (20–30 nm in size, at doses of 100–500 mg/kg) administered intragastrically accumulate in the ovaries. There, they induce overproduction of ROS, and they cause depolarization of the mitochondria in granulosa cells. This results in apoptosis occurring, and also in reduced weight of the ovaries, reduced levels of sex hormones, and reduced indices of reproduction [235]. In a similar way, silica nanoparticles (Si NPs, 5–20 nm in size; given at doses of 3–10 mg/kg, administered orally for eight weeks) induce oxidative stress in the ovaries of C57BL/6 mice. This is characterized by increased levels of ROS and MDA, by decreased activity of SOD, and by inhibition of the Nrf2/HO-1 signaling pathway. These nanoparticles also induce dysfunction of the mitochondria and they activate autophagy. This occurs via the PI3K/AKT/mTOR and PINK1/Parkin pathways. Additionally, they cause apoptosis via the ATM/p53 pathway. All of this leads to a decrease in the number of follicles, to disorders in the estrous cycle, and to fertility that is reduced [236].

In another study, adult female C57BL/6 mice were orally exposed to ZnO nanoparticles (0–400 mg/kg) for 7 days. The exposure resulted in reduced serum estradiol and progesterone levels, increased atretic follicles, and granulosa cell desquamation. Molecular analyses revealed enhanced expression of oxidative stress-related genes *(Keap1*, *Nrf2*) and ER stress markers (PERK, eIF2α, ATF4), along with elevated MDA levels and decreased SOD activity. Restoration of ovarian function by NAC and salinomycin indicates that ZnO toxicity is mediated by oxidative stress, ER stress, and activation of apoptosis [237].

Additionally, polystyrene nanoparticles (PS-NP, ~ 25 nm, 0.5–144 mg/kg orally for 42 days) induce apoptosis and autophagy, and inhibit PI3K/AKT signaling pathway in the granulosa cells of Kunming (KM) mouse ovaries, leading to reduced oocyte quality and female fertility [21]. In vitro studies on antral follicles have shown that TiO₂ NPs (7–39 nm) are internalized by follicular cells at doses of 25 and 50 µg/mL over 96 h, leading to an increase in the Bax/Bcl-2 mRNA ratio (an apoptosis marker) and changes in the expression of genes related to steroidogenesis, resulting in increased progesterone and testosterone levels. This suggests that nanoparticle internalization may disrupt follicular hormonal functions [238].

Taken together, these studies clearly demonstrate that the female reproductive system is highly susceptible to the effects of various nanoparticles. The mechanisms of toxicity include oxidative stress, mitochondrial dysfunction, activation of apoptotic and autophagic pathways, and ER stress. These processes lead to disorders in follicular development, reduced oocyte quality, and decreased levels of sex hormones. All of this, in the end, affects fertility and the normal development of gametes in vertebrates (Fig. 9). These findings emphasize how important it is to do further research into the mechanisms of nanotoxicity and also into the risks that are associated with exposure to nanoparticles when it comes to the reproductive health of women.

The female reproductive system plays a key role in gamete production and early stages of embryonic development, and the proper functioning of oocytes and ovarian follicular cells is essential for vertebrate reproduction. In their study, Chen et al. evaluated the effect of Ag NPs on the maturation of zebrafish (*Danio rerio*) oocytes in vitro. The researchers found that Ag NPs induce germinal vesicle breakdown (GVBD), which is a morphological indicator of resumed meiosis, and decrease cAMP levels in oocytes. A reduction in cAMP levels is a key signal that triggers oocyte maturation. Observations that were done microscopically and ultrastructurally showed that Ag NPs have the ability to induce apoptosis in the follicular cells of the ovary. This is characterized by condensation of the nucleus, fragmentation of the cytoplasm, and vacuolization happening in the mitochondria. Analysis of gene expression showed increased regulation of caspase 3 and caspase 9. There was also increased regulation of genes that are related to oxidative stress, such as *hsp70*, *mt2*, and *gst2*. This suggests that the mechanism of Ag NP toxicity in ovarian follicular cells involves oxidative stress and the activation of the apoptosis pathway. These results highlight the sensitivity of oocytes and follicular cells to Ag NPs. They also indicate a potential risk to reproductive function in vertebrates [239]. In a study by Yang et al., pregnant ICR mice received oral TiO₂ NPs (21 nm, 150 mg/kg) daily from day 8 to day 21 of pregnancy (GD8-GD21). This exposure led to disorders that were permanent and neurobehavioral in the mothers. These disorders included reduced locomotor activity, limited exploration, and decreased responses to new stimuli in the open-field test. The authors linked these impairments to damage that happened in the gut-brain axis. This was shown by changes in the gut microbiota, a decrease in the number of gut neurons, reduced levels of diamine oxidase (DAO), and impaired expression of peptides related to the gut-brain connection. This indicates that the neurotoxicity mechanism is complex and is mediated by the digestive system [240]. In summary, toxicity during pregnancy results not only from the direct transfer of NPs to the fetus, but also from mechanisms that are indirect. These include dysfunction of maternal tissues, modulation of the placental secretome, oxidative stress, and inflammation. The key factors that determine the toxic effects include the route of administration, the size and surface modification of the NPs, the stage of pregnancy, and the mother’s condition.

### Nanoparticle-induced placental and fetal toxicity

**Fig. 10:**
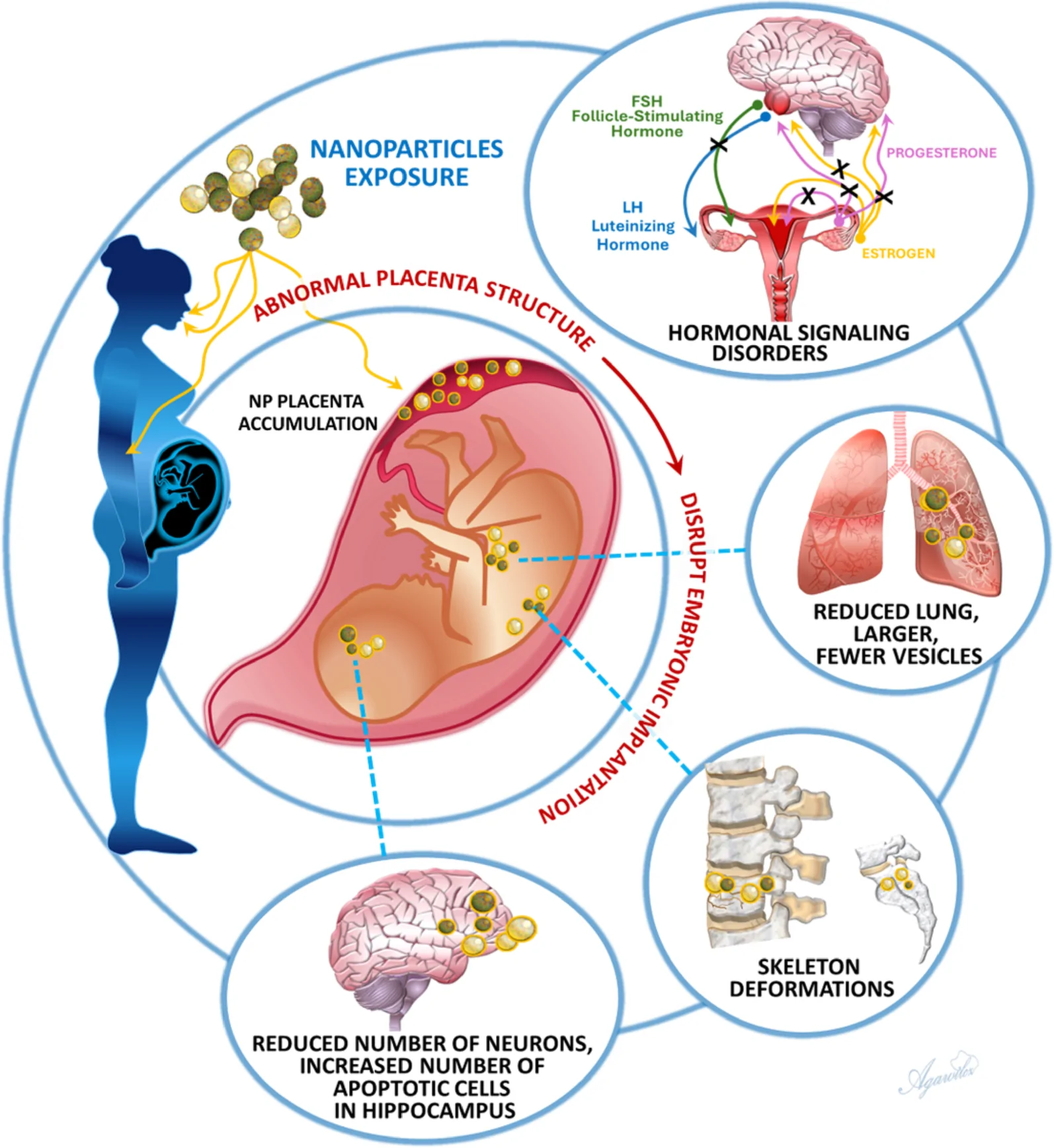
Representation of maternal nanoparticle exposure, placental transfer, hormonal disruption, and resulting fetal developmental toxicity. Maternal exposure to nanoparticles may result in their transplacental transfer and accumulation in placental and fetal tissues, thereby disrupting placental homeostasis and fetal development. The presented pathways include disruption of endocrine signaling, inflammatory activation, oxidative stress, altered placental angiogenesis, trophoblast dysfunction, and impaired vascular architecture. Depending on nanoparticle physicochemical properties, dose, and exposure duration, these alterations may contribute to fetal growth restriction, developmental abnormalities, embryo resorption, and adverse pregnancy outcomes

The field of reproductive toxicology focuses on assessing the impact of chemicals and nanomaterials on reproductive functions and the development of offspring. It includes the analysis of disorders that may possibly occur at any stage of the reproductive process. This means from gametogenesis and fertilization all the way to embryonic development and to pregnancy itself. In the case of nanoparticles, their ability to penetrate barriers that are biologically important, such as the blood-placenta barrier, is of particular importance. This is because it can disrupt the normal development of the embryo and of the fetus. When the parental organism is exposed to nanomaterials during reproduction, this can lead to toxicity in development. This toxicity includes oxidative stress, reactions that are inflammatory, changes that are genetic and epigenetic, and also disturbances in how the hypothalamic-pituitary-gonadal axis is regulated (Fig. 10). The effects that come from these disorders may include fertility that is reduced, problems with implantation, miscarriages, growth retardation inside the uterus (which is called IUGR), birth that is premature, and also defects in development [23].

The studies collected in the review by Teng et al. indicate that the direct penetration of nanoparticles through the placental barrier is one of the main mechanisms of fetotoxicity caused by nanomaterials (Fig. 10). Various types of particles, such as carbon CB and CNTs, polystyrene PS [24], silica SiO₂ [241], metal oxides TiO₂ [24], ZnO [26], ZrO₂ [27], CeO₂, Au [242], silver Ag, and QDs [243] can cross the placental barrier and affect fetal development. The efficiency with which nanoparticles are transferred depends on their physicochemical properties and the physiological condition of the mother. The key factors here include particle size, any surface modifications (for example, when carboxylate groups are more common than PEG), surface charge, and the material the nanoparticles are made from. Studies have shown that nanoparticles, which are spherical in shape, including Au, SiO₂, and ZnO [244], exhibit transfer that is dependent on their size. Furthermore, when appropriate coatings are applied, they can limit the extent to which they penetrate the fetus [233]. Transplacental transport mechanisms include passive diffusion, active transport, and endocytosis [245], including clathrin- and caveolin-dependent processes. In the BeWo b30 in vitro model (Transwell system comprising two chambers separated by an atrophoblast layer membrane, which allows quantification of NPs permeability and analysis of transport mechanisms [245]), translocation of charged polystyrene (PS) particles (50 nm) was primarily dependent on passive diffusion [246]. In contrast, in ex vivo human placental perfusion, PS particles (50–300 nm) move in both directions, suggesting the involvement of active energy-requiring processes [247]. Additionally, maternal exposure to 50 nm AuNPs or 16 nm ZrO₂ increased the activity of the clathrin- and caveolin-dependent endocytic pathway. This supports the hypothesis that transcytosis and mechanisms involving receptors are involved in the transfer of nanoparticles to the fetus [27]. When observations from different research models (in vitro, ex vivo, and in vivo) are combined, this underscores that both the physicochemical properties of NPs and the maternal condition determine whether NPs can cross the placental barrier. It also determines the extent to which they can affect the development of the fetus. And this is crucial for assessing the risk to development.

After crossing the placental barrier, NPs gain access to placental and fetal tissues, where they trigger molecular cascades leading to toxic developmental effects. Numerous studies have documented that prenatal exposure to NPs induces oxidative stress, an inflammatory response, DNA damage, apoptosis, autophagy disorders, and modulation of angiogenesis in the placenta. These effects that depend on the physicochemical properties of the particles and the condition of the mother’s body [233].

In a pioneering study, Zhang et al. showed that exposure of pregnant CD-1 mice to TiO₂ NPs (1 mg/kg/day administered orally from day 1 to day 13 of gestation (GD) led to a decrease in the placental-to-fetal weight ratio, inhibition of vascularization of the placental labyrinth zone, and increased trophoblast apoptosis. This was accompanied by changes in the expression of genes regulating proliferation, such as *Hand1*,* Esx1*,* Eomes*, and caspase-3 activation [248]. In their study, Li et al. showed that exposure of pregnant mice to 100 nm TiO₂ NPs led to impaired placental vascular development, disrupted the vascular architecture of the labyrinth zone, and activated the EndMT (endothelial-to-mesenchymal transition) pathway, indicating that NPs may directly affect placental angiogenesis [249].

At the cellular and molecular levels, engineered nanoparticles such as TiO₂ and SiO₂ can induce ROS overproduction, leading to mitochondrial dysfunction and lipid peroxidation. This, in turn, can activate the NLRP3 inflammasome, as described in mechanistic studies [250]. Adams et al. emphasize that oxidative stress is one of the main mechanisms of placental cell response to the presence of NPs [251].

Autophagy dysfunction in trophoblasts is also considered an important intermediate mechanism of prenatal toxicity. Chen et al. indicated that excessive autophagy activation induced by NPs or oxidative stress can inhibit trophoblast migration and invasion, leading to abnormal placental structure [252]. Excessive activation of this process results in the degradation of key organelles and cellular proteins, thereby limiting placental growth and function and, consequently, potentially leading to fetal growth restriction and neonatal encephalopathy [252, 253].

In addition, nanoparticles can affect the placenta’s endocrine and vascular functions. Maternal exposure to various types of NPs disrupted sex hormone signaling (including LH, FSH, and estradiol), and modulated placental vascular function, resulting in fetal growth retardation, increased embryo resorption, or preterm birth. Such effects have been described for Ag NPs, Au NPs, and TiO₂ NPs, among others, with the degree of toxicity depending on particle size, surface coating type, and dose and duration of exposure [22, 233, 249, 254] (Fig. 10).

Research by Blum et al. provided some of the first evidence that metal nanoparticles can affect hormonal functions and placental morphology. In a model of pregnant CD-1 mice, it was shown that inhalation exposure to cadmium oxide nanoparticles (CdO NPs) at concentrations of 100 and 230 µg/m³ (2.5 h per day on 4.5–16.5 days post-copulation (dpc)) led to a decrease in 17β-estradiol concentration by approximately 50%, disturbances in estrogen receptor expression - an increase in ERα and a decrease in ERβ in the uterus - and morphological changes in the placenta. This was accompanied by delays in female weight gain, reduced placental weight, and restricted fetal growth, suggesting that CdO NPs may indirectly disrupt embryo implantation and development through hormonal dysregulation and modulation of estrogen signaling [255].

Similar effects were observed in studies by Yamashita et al., who showed that intravenous exposure of pregnant mice to silica nanoparticles (SiO₂, 70 nm; 0.8 mg/mouse) and titanium oxide nanoparticles (TiO₂, 217 nm) led to their accumulation in the placenta, liver, and brain of the fetuses, as well as to a reduction of the spongiotrophoblast layer by approximately 50% and increased trophoblast cell apoptosis. A decrease in uterine weight (~ 20%) and fetal weight (~ 10%) was also observed, as well as an increase in the frequency of embryo resorption. Importantly, larger SiO₂ particles (300–1000 nm) and fullerene C₆₀ did not cross the placental barrier or cause significant developmental changes [241].

The collected data suggest that metal nanoparticles and metal oxides can disrupt embryonic development through endocrine disruption, damage to the placental architecture, and induction of trophoblast apoptosis. Furthermore, the work of Dugershaw-Kurzer et al. showed that exposure of human placental explant fragments maintained ex vivo in laboratory conditions, but retaining the structure, viability, and physiological functions characteristic of the placenta in vivo, to SiO₂ and TiO₂ NPs. This exposure caused changes in the secretion of placental proteins associated with angiogenesis, metabolism, and hormone signaling, as well as the induction of antiangiogenic effects in vascular models [256].

### Indirect maternal effects on fetal development

Pregnancy is a period of heightened susceptibility to external factors, including NPs. This is because of numerous changes that happen in physiology, metabolism, and the immune system. These changes can significantly alter how NPs move through the body (their pharmacokinetics) and how available they are (their bioavailability). Teng et al. describe a system that includes the mother, the placenta, and the fetus. In this system, nanoparticles can cause direct and indirect toxic effects. The indirect effects happen through dysfunction of the placenta, through responses that are inflammatory, and through oxidative stress [233]. The toxic effects of nanoparticles during pregnancy can be direct, when particles enter the mother’s bloodstream and cross the placental barrier. It can also be indirect, when they affect the mother’s organs (e.g., lungs, liver, and immune system), leading to the release of inflammatory mediators and ROS, which can disrupt fetal development [244]. Carbon nanomaterials such as CB, carbon nanotubes (SWCNTs, MWCNTs), and fullerenes are used as a model for prenatal toxicity studies due to their widespread use and the often strong inflammatory response in the mother, which indirectly affects the fetus [233].

In C57BL/6 mice, inhalation or intratracheal exposure to CB (~ 14 nm, 42 mg/m³, 1 h/day, GD8-GD18) initiated an inflammatory response in the mother’s body [257]. Similarly, both single intraperitoneal doses (2–5 mg/kg) and intratracheal administration of CNTs (3–5 mg/kg, GD9) caused morphological and skeletal defects in fetuses, including shortened tails, rib adhesions, and vertebral malformations [258]. Functionalization of NP surfaces, e.g., with amino groups or PEG, increased toxicity when amino-functionalized SWCNTs were administered intravenously (0.1, 10, 30 µg/mouse, GD6) at multiple doses, leading to increased fetal resorption and skeletal deformities. Qin et al. showed that MWCNTs can adsorb endocrine-disrupting compounds, such as dibutyl phthalate (DBP), thereby synergistically increasing developmental toxicity and causing endocrine disruption in fetuses [259]. Metal and metal oxide nanoparticles, including ZnO and TiO₂, show toxic potential in pregnancy models. Oral exposure to ZnO NPs (GD6-15) in mice resulted in delayed fetal growth, increased resorption, reduced number of live fetuses, and skeletal deformities [233]. Intravenous injection of ZnO NPs (44 nm, 5, 10, 20 mg/kg, GD6-GD20) resulted in increased numbers of dead fetuses, post-implantation losses, and reduced fetal weight at the highest dose of 20 mg/kg. Similarly, TiO₂ NPs administered intravenously, by inhalation, or orally increased the resorption rate, reduced uterine and fetal weight, and induced skeletal deformities [260, 261]. A study by Bideskan et al. showed that prenatal and lactational exposure of female Wistar rats to TiO₂ NPs (~ 21 nm, 100 mg/kg/day, oral route) led to an increased number of apoptotic cells and a reduction in neurons in the hippocampus of offspring (Fig. 10). Immunohistochemical analysis revealed that there was an increase in the expression of the protein that is proapoptotic, which is called BAX. This protein promotes the release of cytochrome *c* from the mitochondria. This then initiates the caspase cascade, leading to the controlled death of neurons. At the same time, expression of the anti-apoptotic protein Bcl-2 decreased. This protein normally stabilizes the mitochondrial membrane and inhibits this cascade from occurring. All of this indicates that the mitochondrial pathway for apoptosis was being activated [262]. Research performed by Dugershaw-Kurzer et al. using an ex vivo model of the human placenta showed that TiO₂ NPs of around 30 nm in size and SiO₂ NPs of around 50 nm in size, when present at concentrations of 10 to 50 µg/mL, have the ability to modulate the secretory activity of the placenta, which is called its secretome. They do this by affecting angiogenic factors, metabolism, and hormone production. This can then lead to disorders in the development of the fetus. And all of this can happen even without large amounts of particles needing to cross the placental barrier [256].

The lungs are also among the key targets of nanomaterials’ toxic effects during the prenatal period. Studies conducted by Paul et al. showed that exposure of pregnant C57BL6/J female mice to Ag, TiO₂, and CeO₂ nanopowders (spherical, 10 nm, coated with poly(vinylpyrrolidone) (PVP)) leads to permanent disturbances in the development of the offspring’s lungs (Fig. 10). An increase in the mean linear dimension of the alveoli was observed in fetuses and newborns, indicating enlarged, underdeveloped alveolar structures and a decrease in their number compared to the control group. These changes indicate impairment of alveolarization. This means the process by which alveoli form and mature in lungs that are still developing. These changes also persisted after birth. This indicates that the damage to the structure was irreversible. When we look at the level of the molecules, it has been shown that exposure to nanoparticles causes a decrease in how genes that are key for angiogenesis and for the remodeling of the lungs are expressed. Among these genes, we can find vascular endothelial growth factor-α (VEGF-α), matrix metalloproteinase 9 (MMP-9), and fibroblast growth factor-18 (FGF-18). At the same time, concentrations of anti-inflammatory cytokines decreased. Examples include GM-CSF, IL-10, and IL-15. In the groups that were exposed to CeO₂ and to Ag NPs, significant reductions were observed in the levels of VEGF that were in the amniotic fluid. There was also an accumulation of silver seen in the lung tissue of the fetus. This confirms that these NPs can cross the placental barrier and bioaccumulate. The results indicate that exposure to metallic nanoparticles before birth disrupts signaling pathways responsible for angiogenesis and epithelial cell differentiation in the alveoli. This results in permanent impairment of lung maturation. The lungs can therefore be considered one of the key organs targeted by nanofetotoxicity, and they are particularly vulnerable to the effects of metallic nanomaterials [263].

In summary, nanoparticle toxicity during the prenatal period results from a combination of factors. These include direct effects on fetal tissues, placental dysfunction, and disruptions to signaling pathways in cells and the endocrine system. A comprehensive understanding of the physicochemical properties of nanoparticles and their interactions with the mother’s organism is essential for accurately assessing the risks before birth. It is also essential to develop effective strategies to safeguard the fetus’s health.

## Regulatory aspects of nanomedicinal products

The regulatory framework for nanotechnology-based medicinal products has continued to evolve, although neither the European Union nor the United States has adopted a single, self-standing legal regime dedicated exclusively to nanomedicines. The entities such as the European Medicines Agency (EMA) and the Food and Drug Administration (FDA) manage these products and medical devices. At the same time, they are developing guidelines, reflection papers, and reports. The purpose is to provide initial guidance on how nanomedicines should be developed and evaluated.

### European union regulatory framework

There is no EU regulatory framework dedicated exclusively to nanomedicines. Instead, nanomedicines are primarily assessed under the general medicinal-products framework and the main laws that govern this area especially include the following: : (i) Directive 2001/83/EC, consolidated version 01/01/2025 [264], (ii) Regulation (EC) No 726/2004, current consolidated version 28/01/2022, relating to medicinal products for human and for veterinary use [265]. Where the product is a medical device, a borderline product, or a medicine used in combination with a medical device, Regulation (EU) 2017/745, current consolidated version 01/01/2026, on medical devices and the EMA/CMDh Q&A guidance on the implementation of the MDR and IVDR are also relevant [266]. The 2024 amendment introduced by Regulation (EU) 2024/1860 should also be noted, particularly in relation to the gradual roll-out of Eudamed, supply-interruption obligations, and transitional provisions [267].

The European REACH (Registration, Evaluation, Authorisation and Restriction of Chemicals) regulation includes provisions for nanomaterials, ensuring their safety evaluation before being placed on the market. The REACH framework, Regulation (EC) No 1907/2006, consolidated version 23/10/2025, should be understood as a complementary chemicals-regulation regime rather than as the principal marketing-authorization pathway for nanomedicinal products [268]. Its relevance lies mainly in the registration, information, and chemical-safety obligations applicable to substances and nanoforms. Commission Regulation (EU) 2018/1881 amended several REACH annexes to address nanoforms of substances, and the current consolidated version of REACH should therefore be cited when discussing nanoform-specific data requirements [269]. It also imposes obligations for registration and risk assessments for nanomaterials. Under these regulations, producers and importers of nanomaterials must provide detailed information. This information must cover the characteristics of the nanomaterials and their potential effects on health and the environment. EU published a revised recommendation 2022/C 229/01 for the definition of the term nanomaterial [270]. The European Chemicals Agency and European Food Safety Authority provide oversight of nanomaterials, particularly in relation to consumer safety and environmental impact [271].

### European medicines agency (EMA)

The EMA has established a robust regulatory framework that addresses the unique challenges of nanomedicine identity and biological performance. Rather than focusing solely on the active pharmaceutical ingredient (API), these documents emphasize the nanostructure itself as a critical determinant of safety and efficacy. At the scientific-regulatory level, the EMA/HMA EU-IN Horizon Scanning Report on nanotechnology-based medicinal products for human use, titled “Nanotechnology-based medicinal products for human use” EMA/20,989/2025/Rev.1, published in January 2025, provides the most current EU-level synthesis [272]. The aim of this document is to support the development of pharmaceuticals and to help inform how clinical and non-clinical studies are designed.

The document indicates that there is no specific and appropriate EU regulatory framework for nanomedicines exists, and that existing EMA reflection papers on liposomal products, intravenous iron-based nano-colloidal products, block-copolymer micelles, surface coatings, and generic nanoparticle iron medicinal products should continue to apply. Some examples of these are: (i) A reflection paper about the data that is required for products that are liposomal and given intravenously, when they are developed with reference to an innovator product that is also liposomal [273]. (ii) Reflection paper on surface coatings: general issues for consideration regarding parenteral administration of coated nanomedicine products [274]. (iii) Reflection paper on the data requirements for intravenous iron-based nano-colloidal products [275]. (iv) Joint MHLW/EMA reflection paper on the development of block copolymer micelle medicinal products [276]. These documents collectively address the challenges of nanomedicines, in which traditional bioequivalence simply measuring blood concentrations is insufficient to guarantee therapeutic equivalence. Nanomedicine products need to be physicochemically characterized in terms of particle morphology (average size, polydispersity), surface properties, drug loading, and drug state. Additionally, the authors emphasize that stability and release kinetics (including in vitro release and interaction with plasma proteins), as well as both total encapsulated and unencapsulated drug content, should be considered. Unique safety risks and immunogenicity are particularly associated with the surface coatings of nanomedicine. The authors highlight that bioequivalence must be demonstrated with high precision. A combination of rigorous physicochemical and multi-modal pharmacokinetics studies is required to prove the efficacy and safety of nanodrugs. At the same time, the report recommends strengthening regulatory expertise, improving tracking of nanomedicines in regulatory submissions and The Clinical Trials Information System (CTIS), and considering updates to guidance through a risk-based, product-specific approach.

One critical challenge is that robust regulations cannot be established without input from the people who develop products and from the scientists researching the effects of nanomedicines. The EU-IN report on horizon scanning acknowledges that without this kind of collaboration, the people who make regulations will not be able to close the knowledge gap to achieve comprehensive, reliable regulations. For this purpose, the European Innovation Network (EU-IN) was established. Its goal is to help stakeholders collaborate and enhance expertise in regulation. People who apply are encouraged to contact their National Competent Authorities to become involved in this process (EMA/20989/2025, pages 6 and 7).

It is also important to note that several medical products containing nanoparticles have already been approved in the EU and the US. For example, the EMA issued guidance specific to certain products on bioequivalence. This was for pegylated liposomal doxorubicin hydrochloride [277] and also for liposomal amphotericin B [278]. In a similar way, the FDA released guidance that was in draft form for doxorubicin hydrochloride [279] and for amphotericin B [280]. These contain recommendations that are not binding. What is significant is that most of the approved nanomedicines are based on active substances already authorized under Directive 2001/83/EC.

### Food and drug administration (FDA)

In the United States, FDA has likewise not adopted a single legally binding nanomedicine-specific framework indicating the limits of use. However, the FDA has issued non-binding guidance intended for the industry. Some examples of this guidance include the following: (i) a document called “Considering whether an FDA-regulated product involves the application of nanotechnology” remains an overarching nonbinding framework for identifying FDA-regulated products involving nanotechnology [281]; and (ii) 2022 final guidance called “Drug Products, including biological products, that contain nanomaterials” [282] and the 2021 final guidance on liposome drug products, which address product characterization, manufacturing controls, safety evaluation, testing, stability, pharmacokinetics, bioavailability, and labeling [283]. These documents do not establish new legal requirements but outline how existing regulations should be interpreted for products containing nanomaterials. The first provides a framework for determining if a product involves nanotechnology and defines relevant terms. The second offers recommendations for developing human drug and biological products containing nanomaterials, covering aspects such as product characterization, manufacturing controls, safety evaluation, testing methodologies, and stability. Beyond the examples mentioned above, the FDA has also issued a final guidance on liposomal drug products. This guidance covers various areas, such as the chemistry involved, the controls used in manufacturing, how the drug moves through the human body and its bioavailability, and what documentation is needed for labeling [284]. This guidance discusses the information that people who apply should submit when filing a New Drug Application (NDA) or an Abbreviated New Drug Application (ANDA) for a liposomal drug product. Additionally, the FDA publishes documents explaining the risks and updates to products approving nanomaterials [285].

A comparison of the regulatory approaches used by the EMA and the FDA for medical products based on nanotechnology is presented in Table 4.

**Table 4:** Comparison of the regulatory frameworks employed by the EMA and the FDA

Feature	EU/EMA	FDA
Acts of law that are applicable	Directive 2001/83/EC consolidated version 01/01/2025 Regulation (EC) No 726/2004 consolidated version 28/01/2022 Regulation (EU) No 2017/745 on medical devices, consolidated version 01/01/2026, as amended intr alia by Regulation (EU) No 2024/1860 Regulation (EC) No 1907/2006 (REACH) consolidated version 23/10/2025 Commission Regulation (EU) 2018/1881 addressing nanoforms of substances Commission Recommendation 2022/C229/01 on the definition of nanomaterials	Federal Food, drug and Cosmetics Act, 21 U.S.C. § 301 et seq., especially 21 U.S.C. § 399e [286]
Reflection paper/guidances	4 reflection papers, 1 scanning report	2 nonbinding guidance for industry and 1 for liposome drug products
Product-specific guidances	Pegylated liposomal doxorubicin hydrochloride Liposomal amphotericin B	Draft guidance on doxorubicin hydrochloride Draft guidance on amphotericin B

## Pharmacovigilance

**Fig. 11:**
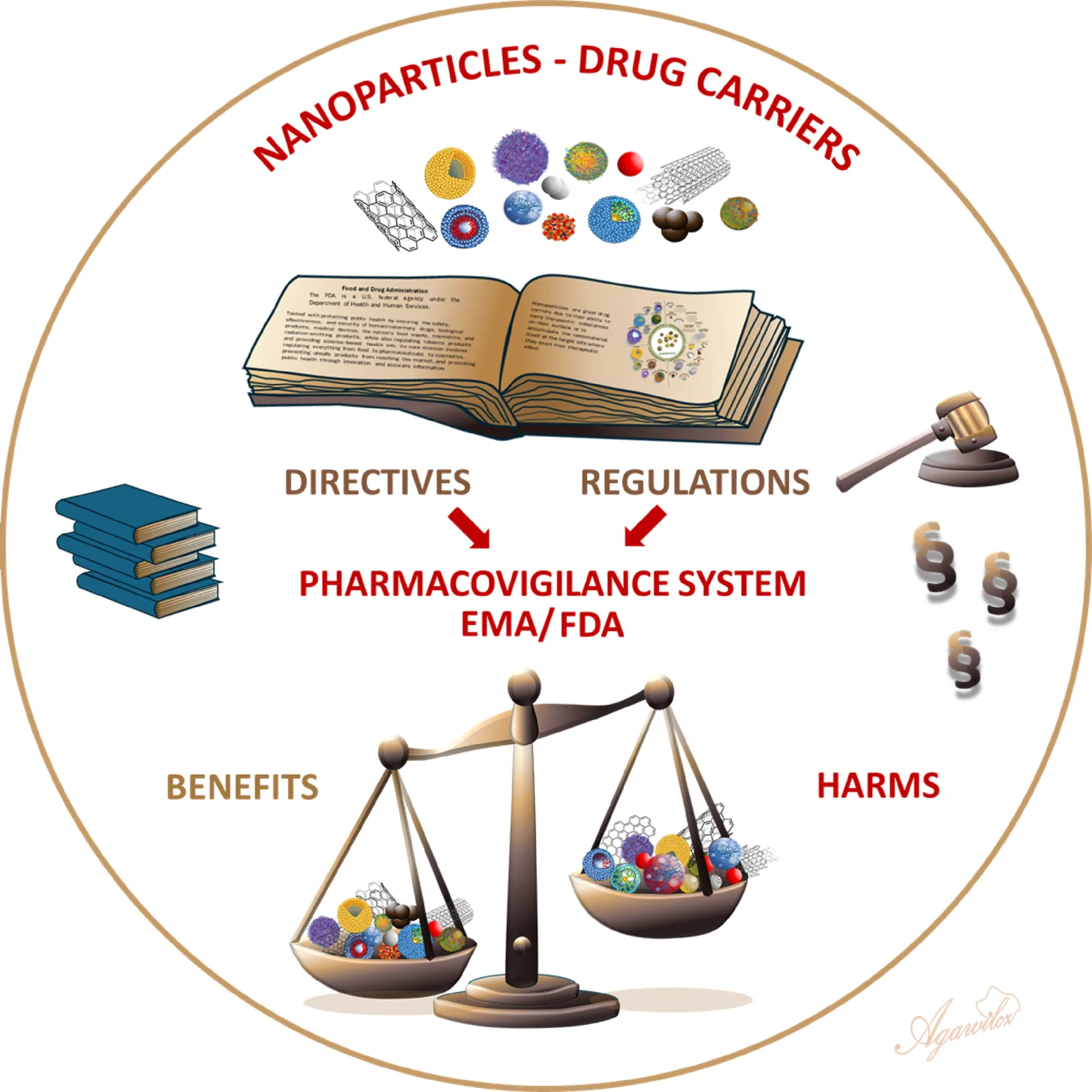
Conceptual representation of regulatory oversight and the balance between benefits and risks of nanomedicines, underlying the need for enhanced pharmacovigilance. The figure illustrates the relationship between the therapeutic benefits of nanomedicines and the potential risks associated with their unique physicochemical properties, emphasizing the importance of regulatory oversight and post-marketing safety monitoring. Due to their complex biological interactions, nanomedicines may exhibit pharmacokinetic, biodistribution, and toxicity profiles that differ from those of conventional pharmaceuticals. The presented concept highlights the role of pharmacovigilance systems, risk management strategies, and continuous safety assessment in supporting the safe clinical implementation of nanoparticle-based therapeutics

An important matter worth mentioning is the regulation of registration and reporting of side effects for nanomedicines. Given that nanomedicines may behave differently from conventional pharmaceuticals due to their nanoscale properties, regulators require greater vigilance in post-marketing monitoring (Fig. 11).

### EMA pharmacovigilance

Nanomedicines are subject to the EU pharmacovigilance legislation, including the above-mentioned Directive 2001/83/EC and Regulation (EC) No 726/2004. Adverse drug reactions (ADRs) are reported through EudraVigilance – a centralized, European database of suspected adverse drug reaction reports [287]. Marketing authorization holders (MAHs) are required to submit reports to this database in line with current legislation. The EMA’s reflection papers on nanomedicines outline specific considerations for pharmacovigilance and highlight the need to tailor safety and toxicity assessments. Furthermore, when applying for a new marketing authorization in the EU, companies must submit a Risk Management Plan (RMP). These plans are continually updated and amended throughout the medicine’s lifestyle as new information becomes available [21]. Regular periodic safety update reports (PSUR) [288] and renewal evaluations (which may include updated RMPs submitted alongside a PSUR) help to reassess risks. Thus, post-marketing safety reporting comprises EudraVigilance and PSUR. It is important to highlight that RMPs are a standard requirement in the EU.

### FDA pharmacovigilance

The agency responsible for pharmacovigilance within the FDA is the Center for Drug Evaluation and Research (CDER). This center oversees the safety and effectiveness of pharmaceutical products. This includes activities related to surveillance after products have been put on the market. As with the EMA framework, there is no specific US regulation for nanomedicines at present. Because of this, they must follow the existing processes for Investigational New Drug (IND) applications and New Drug Applications (NDA). For certain higher-risk products, this may include the submission of a Risk Evaluation and Mitigation Strategy (REMS) program [289]. The FDA maintains a database analogous to the EMA’s EudraVigilance, called the FDA Adverse Event Reporting System (FAERS) [290]. In addition, the FDA provides a reporting tool called MedWatch that allows healthcare professionals and consumers in the United States to report adverse events (including adverse drug reactions), medication errors, and product quality problems. MedWatch is a continuous reporting system. FAERS, on the other hand, is a web-based tool used to collect, store, and analyze adverse event reports submitted by healthcare professionals, consumers, and manufacturers. It should be noted that while various parties can report FAERS and MedWatch, PSURs can be submitted only by drug manufacturers. It should also be emphasized that nanomedicines in the US are subject to standard post-marketing surveillance; there is no exclusive law for nanotechnology products or nanomedicines. However, the FDA has acknowledged that nanomedicines have unique features. It is reflected in guidance documents, such as the one titled “Considering Whether an FDA-Regulated Product Involves the Application of Nanotechnology”. This document came out in June 2014. In this guidance, the FDA emphasizes that it “*will regulate nanotechnology products under existing statutory authorities*,* in accordance with the specific legal standards applicable to each type of product under its jurisdiction*” [291]. The guidance also encourages manufacturers of these products to consider the unique properties of nanomaterials when conducting safety assessments. It also encourages them to consult with the FDA early, while the product is still under development. The rules for reporting side effects for nanodrugs at the EMA and FDA are included in Table 5.

**Table 5:** Regulation of registration of side effects for nanocarriers

Feature	EMA	FDA
Reporting System	EudraVigilance, a centralized database	FAERS, MedWatch
Responsible body	The pharmacovigilance risk assessment committee (PRAC),	Center for Drug Evaluation and Research (CDER)
Specialised monitoring for nanomedicines	Yes, according to the reflection papers	Yes – according to guidelines
Submission body	Marketing authorization holders (ADRs), also known as manufacturers; consumers can report only via the national authority	Both healthcare professionals and consumers (MedWatch), as well as manufacturers (FAERS)
Risk plan	Risk Management Plan (RMP) - obligatory	Risk Evaluation and Mitigation Strategy (REMS)
Post-Market Reports	Periodic safety update reports (PSUR)	via MedWatch, adverse event reports

## Challenges and future perspective

The increasing demand for effective and affordable healthcare solutions has driven the rapid development of nanomaterials for drug delivery and medical devices [292]. Conventional therapeutic approaches are often limited by poor stability, low bioavailability, inadequate target specificity, and systemic side effects [293]. In this context, nanotechnology offers promising opportunities for the development of advanced drug delivery systems and next-generation pharmaceutical formulations, enabling improved drug stability, prolonged shelf-life, enhanced thermoresistance, and better resistance to environmental conditions, while potentially reducing required dosages and distribution costs [294]. As a result, the application of nanotechnology in biomedicine continues to expand, driven by its potential to improve therapeutic outcomes. Despite these advantages, significant scientific and translational challenges still hinder clinical implementation. Key issues include limited challenges in large-scale, reproducible manufacturing, targeting efficiency, and unresolved long-term safety concerns. Future research should therefore focus on improving nanoparticle design for more precise targeting, enhancing production scalability and reproducibility, and ensuring comprehensive safety evaluation. In addition, the integration of personalized medicine strategies, alongside improved nanomaterial characterization, predictive preclinical models, and robust toxicity assessment frameworks, will be essential for the safe and effective clinical translation of nanotherapeutics.

### Large-scale and reproducible manufacturing

Although many nanoparticle formulations demonstrate promising preclinical outcomes, large-scale manufacturing remains a major barrier to clinical translation [295]. Key challenges include reproducibility, batch-to-batch consistency, physicochemical stability, and cost-effective production. Moreover, the clinical implementation of nanotechnology raises important safety and environmental concerns. Due to their small size and high biological reactivity, nanomaterials can cross biological barriers and induce adverse effects, including tissue damage, inflammation, and systemic toxicity following accumulation in vital organs [296]. In parallel, the increasing scale of nanomaterial production heightens the risk of unintended environmental release, underscoring the need for safer and more sustainable manufacturing strategies [297].

Environmental sustainability is also a critical issue, as conventional top-down approaches often generate significant waste, while some bottom-up methods require high energy input or involve toxic reagents [298]. On the other hand, production of green-synthesized nanomaterials is often limited by low yields, non-uniform particle sizes, complex extraction processes, and variability in raw material availability depending on season and region. Precise control of physicochemical properties remains essential, as even minor variations during synthesis can significantly alter nanomaterial performance. Accordingly, a comprehensive characterization of both manufacturing processes and final products is required to ensure stability, quality, and regulatory compliance. Therefore, the development of green, energy-efficient, and solvent-free synthesis strategies is essential. Methods such as microfluidics, flash nanoprecipitation, porous and solid hollow-fiber cooling crystallization systems, and certain supercritical CO₂ (scCO₂)-based approaches, including SAILA, SEA-PES, and SFEE, are currently applied in batch or semi-batch configurations but have strong potential for adaptation to continuous manufacturing [299]. Emerging technologies, including automated manufacturing systems, AI-driven process optimization, and computational modeling approaches, can further support the transition toward continuous manufacturing by enhancing scalability, reproducibility, and process control [300]. Future development should focus on strengthening academia–industry collaboration to accelerate translational progress and align research with clinical and industrial needs. In effect, scalable and sustainable manufacturing platforms should be arranged, supported by pilot-scale validation. Moreover, harmonization of international standards and ISO guidelines will be critical for improving reproducibility and facilitating clinical translation [301].

### Regulatory and safety considerations

Future progress in this field will require the establishment of standardized regulatory frameworks and harmonized characterization protocols for nanomedicines. Comprehensive physicochemical characterization, including particle size distribution, polydispersity index (PDI), zeta potential, morphology, surface chemistry, aggregation behavior, and drug release kinetics, remains essential for ensuring reproducibility, stability, and clinical safety [302]. Another important limitation in current research is the insufficient deconvolution of the individual contributions of nanosystem components. Most studies focus on the overall performance of the final formulation, while the role of specific structural elements, such as core nanoparticles, shell architectures, surface coatings, active agents, targeting ligands, and functionalization molecules-is often not systematically assessed. This makes it difficult to attribute observed effects to distinct components and limits the development of reliable structure–activity and structure–toxicity relationships. In effect, studies should therefore include systematic comparative analyses of unmodified nanoparticles, core–shell systems, and surface-functionalized nanocarriers, both empty and drug-loaded, to better understand how each component contributes to biological performance and therapeutic efficacy, and to determine toxicity profiles. Consequently, establishing the safety and therapeutic efficacy of nanomedicines necessitates a robust framework of rigorous physicochemical characterization alongside multimodal pharmacokinetic evaluations.

In this context, machine learning (ML) approaches may become increasingly important tools for nanotoxicology and regulatory assessment [303]. Conventional experimental methods for cytotoxicity evaluation are often costly, labor-intensive, and time-consuming, particularly when large libraries of nanomaterials are under investigation. Recent studies have demonstrated that ML-based models, including random forest regression and classification algorithms, can successfully predict nanoparticle cytotoxicity using physicochemical parameters, cellular characteristics, and experimental conditions. Feature importance analyses demonstrated that nanoparticle composition, concentration, zeta potential, size, exposure time, and experimental conditions strongly influence toxicity prediction [304]. An important parameter to consider is the formation of the protein corona under physiological conditions, as nanoparticle–protein interactions can significantly influence pharmacokinetic parameters, targeting efficiency, cellular uptake, and immune recognition. Standardized approaches for protein corona characterization may improve the predictability of in vivo nanoparticle behavior and therapeutic outcomes. Currently, the integration of ML-based predictive approaches with standardized experimental datasets may significantly improve the analysis of protein corona formation and its relationship with nanoparticle physicochemical properties [305]. Consequently, ML-driven models could accelerate safety assessment, enhance structure–toxicity analyses, and support the development of more reliable regulatory frameworks for nanomedicine applications.

### Optimization of targeting efficiency

The integration of nanotechnology with personalized medicine represents a promising direction in future therapeutics. Patient-specific nanoparticle systems designed based on genetic, molecular, or biomarker profiles may significantly improve treatment precision and therapeutic outcomes [306]. However, achieving efficient and selective targeting remains challenging due to biological complexity and interpatient variability. To address these limitations, smart polymer-based nanocarriers have emerged as an important strategy for enhancing targeting efficiency and controlling drug release [307]. These systems can respond to internal stimuli (e.g., pH, enzymes, redox conditions) or external triggers (e.g., temperature, light, magnetic fields), enabling site-specific and temporally controlled drug delivery. When combined with peptide- or ligand-based targeting, such stimuli-responsive systems further improve delivery precision and therapeutic performance [308].

Artificial intelligence and machine learning approaches are increasingly applied to the rational design of nanocarriers, targeting ligands, and stimuli-responsive materials. In particular, tumor-homing peptides (THPs) are promising targeting moieties for nanoparticle functionalization, but their experimental identification is labor-intensive and costly. Computational platforms such as TumorHPD, THPep, and SCMTHP enable in silico prediction and optimization of THP sequences, supporting the design of improved targeting ligands. More broadly, ML-based models and computational frameworks, including QSAR, PBPK, PK/PD, and agent-based approaches, enable prediction of nanoparticle properties, nano–bio interactions, biodistribution, and toxicity, thereby reducing experimental burden and accelerating translational development [300]. Overall, the convergence of AI-driven design, peptide-based ligands, and smart polymeric nanocarriers represents a key strategy for advancing precision nanomedicine. While these approaches significantly improve design efficiency and functional performance, further work is still required to enhance the reliability of in vivo targeting and to enable clinical translation. Engagement with advanced physiological models, such as organ-on-chip systems and 3D organoid platforms, may improve the prediction of human-relevant biological responses and address limitations of conventional animal models. Finally, safe-by-design principles, regulatory harmonization, and scalable GMP manufacturing strategies will be crucial to bridge the gap between laboratory research and clinical application.

Certain properties of nanomaterials that are typically regarded as drawbacks can be used therapeutically. The induction of the oxidative stress can be reconsidered as a therapeutic advantage when precisely confined to the disease site [309]. In tumor-targeted applications, these effects may be locally amplified to promote selective cancer cell damage while minimizing systemic exposure. In particular, ROS inducers may hinder survival signalling, enhance immunogenic cell death, and increase tumor susceptibility to DNA damage by elevating oxidative stress levels [310]. This shift in perspective enables the transformation of intrinsic nanotoxicity into a controllable, site-specific therapeutic mechanism, particularly when integrated with active targeting and stimuli-responsive delivery systems.

Nanotechnology offers a powerful platform to overcome key limitations of conventional therapeutics, enabling improved drug stability, bioavailability, and targeted delivery. However, clinical translation remains limited by challenges in scalable, reproducible manufacturing, incomplete safety understanding, and insufficient standardization of nanomaterial characterization. In consequence, future progress will rely on the development of sustainable, continuous, and scalable production strategies, alongside harmonized regulatory frameworks and robust toxicity assessment. Additionally, the integration of artificial intelligence, predictive modeling, and advanced in vitro systems is expected to accelerate rational design and safety evaluation.

## Conclusions

Despite the growing body of literature on nanoparticle-induced toxicity, a major limitation remains the difficulty in disentangling the relative contribution of nanoparticle core composition, surface chemistry, and associated compounds to the overall toxicological profile. In most studies, nanoparticles are evaluated as integrated systems, which complicates the interpretation of observed biological effects. Furthermore, variability in physicochemical characterisation and the absence of standardised experimental protocols further limit the capacity for cross-study comparisons. This should be considered when interpreting the toxicological findings discussed in this review.

Nanocarriers represent a major advancement in modern pharmacotherapy. They do this by enhancing the stability, solubility, and targeted delivery of therapeutic agents. However, the same physicochemical properties that underlie their effectiveness may also contribute to biological toxicity. The most frequently reported adverse effect is the generation of reactive oxygen species. They also include the activation of pro-inflammatory cytokines, modulation of the immune response, and accumulation in organs that perform detoxification, such as the liver, kidneys, and spleen. Additionally, they include interactions with enzymes belonging to the cytochrome P450 family. All of this is underscoring how complex their biological behavior actually is.

The available evidence indicates that nanoparticles may exert significant effects on both the male and female reproductive systems. This can lead to impairment of spermatogenesis, oocytes damage, hormonal imbalances, placental dysfunction, and altered fetal development. These effects are mediated by different mechanisms. Examples of these are oxidative stress, dysfunction that happens in the mitochondria, the induction of apoptosis and autophagy, and also the disruption of signaling pathways that are essential for cells to divide and for the regulation of reproductive hormones. What is important here is that the severity of reproductive toxicity depends on the type of nanoparticle, its size, any surface modifications, the dose given, and the stage of development of the organism exposed. This highlights the need for a rigorous risk assessment of reproduction and embryonic development.

Although studies that are preclinical in nature suggest that nanocarriers may be able to reduce the toxicity that drugs cause to the whole system when they are compared to formulations that are more conventional, the effects that they have over the long term on how cells maintain their internal balance and on metabolic processes are still not characterized in a way that is sufficient. More research is going to be required, and this is especially true when it comes to safety regarding reproduction and toxicity to the fetus. This research should include standardized experimental models and detailed analyses of the molecular mechanisms of toxicity in the gonads and the placenta.

In addition, there is increasing evidence that nanoparticles can alter the composition and function of the gut microbiome. This can promote dysbiosis, changes in how short-chain fatty acids are metabolized, and inflammatory responses. It can also disrupt the gut-to-brain and gut-to-liver axes. Because of this, endpoints related to the microbiome should be incorporated into routine toxicology and safety assessments for nanomaterials used in consumer products and pharmaceutical products.

When we look at the whole picture, a comprehensive understanding of how nanocarriers interact with biological systems is essential for clinical applications to be both safe and effective. The continued development in the field of nanomedicine must therefore be accompanied by updated regulatory frameworks and robust pharmacovigilance systems. The purpose is to ensure patient safety and reliable outcomes observed in clinical practice.

In our opinion, the future development of nanotoxicology should focus on improving the understanding of how nanoparticles interact with biological systems and on establishing more consistent methods for evaluating their safety. Current toxicological assessment strategies are still largely based on conventional models developed for small-molecule drugs, which may not fully reflect the unique physicochemical complexity and biological behavior of nanosystems. Therefore, future studies should increasingly integrate physicochemical characterization with biological response profiling, including immune interactions, biodistribution patterns, biodegradation, and chronic low-dose exposure effects.

We also believe that regulatory frameworks should evolve toward more flexible and nanosystem-specific evaluation strategies rather than relying solely on generalized toxicological paradigms. Considering the heterogeneity of nanomaterials, future regulatory decision-making should account not only for chemical composition but also for dynamic properties such as surface functionalization, protein corona formation, aggregation behavior, and route-dependent biological interactions. Greater international harmonization of testing protocols and closer collaboration among researchers, clinicians, industry and regulatory agencies may be essential for establishing reproducible and clinically relevant nanosafety standards.

Importantly, the future of nanomedicine should not be driven exclusively by minimization of toxicity, but rather by achieving an optimal balance between therapeutic benefit and acceptable biological risk. We believe that such an integrated benefit-risk approach will be fundamental for the responsible and sustainable clinical translation of nanoparticle-based therapeutics.

## Data Availability

No datasets were generated or analysed during the current study.

## Declarations

All manuscripts must contain the following sections under the heading ‘Declarations’:

## Abbreviations

af-SWCNTs: Acid-functionalized single-walled carbon nanotubes
Ag NPs: Silver nanoparticles
alkyne-THCPSi: Alkyne-terminated thermally hydrocarbonized PSi
ANDA: Abbreviated New Drug Application
APTES-TCPSi: Aminopropylsilane-modified thermally carbonized porous silicon
ATP: Adenosine triphosphate
Au NPs: Gold nanoparticles
CARPA: Complement activation-related pseudoallergy
CAT: Catalase
CB: Carbon black
CdO NPs: Cadmium oxide nanoparticles
CeO_2_ NPs: Cerium oxide nanoparticles
CTIS: Clinical Trials Information System
CNF: Cellulose nanofibers
CNTs: Carbon nanotubes
CuO NPs: Copper oxide nanoparticles
CYP: Cytochrome P450
DAO: Diamine oxidase
Dpc: Days post-copulation
EMA: European Medicines Agency
EndMT: Endothelial-to-mesenchymal transition
EPR: Enhanced Permeability and Retention
ER: Endoplasmic reticulum
FDA: Food and Drug Administration
Fe_2_O_3_NPs: Iron oxide nanoparticles
FGF-18: Fibroblast growth factor-18
GO: Graphene oxide
GNPs: Graphene nanoplatelets
GQDs: Graphene quantum dots
GR: Graphene
GSH-Px: Glutathione peroxidase
GVBD: Germinal vesicle breakdown
HMDMs: Human monocyte-derived macrophages
IUGR: Intrauterine growth retardation
ML: Machine learning
MMGD: Mannose-functionalized poly(propyleneimine) glycodendrimers
MMP-9: Matrix metalloproteinase 9
MRI: Magnetic resonance imaging
MWCNTs: Multi-walled carbon nanotubes
MWNT: Multi-walled nanotubes
NACOS: N-acetyl chitooligosaccharides
NDA: New Drug Application
NF-κB: Nuclear factor
NK: Natural killer
NLRP3: NOD-like receptor family, pyrin domain-containing 3
NMs: Nanomaterials
ONOO-: Nitrosyl peroxide
OS-PEG-Man: Oligomaltose surface–poly(ethylene glycol)mannose
PDI: Polydispersity index
PDMAEMA: Poly(2-dimethylaminoethyl methacrylate)
PEI: Polyethylenimine
PIMs: Pulmonary intravascular macrophages
PS: Polystyrene
PVP: Polyvinylpyrrolidone
PVP-AgNPs: Ag NPs stabilized with polyvinylpyrrolidone
QDs: Quantum dots
REACH: Registration, Evaluation, Authorisation and Restriction of Chemicals
rGO: Reduced graphene oxide
ROS: Reactive oxygen species
SCFA: Short-chain fatty acid
SiO₂ NPs: Nanosilica
SOD: Superoxide dismutase
SWCNTs: Single-walled carbon nanotubes
SWNTs: Single-walled nanotubes
tau-MWCNTs: Taurine-functionalized multi-walled carbon nanotubes
TCPSi: Thermally carbonized porous silicon
TLR: Toll-like receptor
TNF-α: Tumor necrosis factor
USPIO: Ultrasmall superparamagnetic iron oxide nanoparticles
VEGF-α: Vascular endothelial growth factor-α
ZnO NPs: Zinc oxide nanoparticles
